# Dual‐Pedicle Tissue‐Engineered Trachea Promotes Biomimetic Cartilaginous Framework, Vascularization, and Epithelial Lining for Long‐Segment Tracheal Reconstruction

**DOI:** 10.1002/advs.202514724

**Published:** 2025-10-13

**Authors:** Ziming Wang, Yuming Wang, Zihao Chen, Erji Gao, Liang Guo, Juanjuan Li, Siqiang Zheng, Jiaoyu Yi, Zhe‐Sheng Chen, Bo Tao

**Affiliations:** ^1^ Department of Thoracic Surgery Shanghai Pulmonary Hospital Tongji University School of Medicine Shanghai 200433 China; ^2^ Shanghai Lung Cancer Center Shanghai Chest Hospital Shanghai Jiao Tong University School of Medicine Shanghai 200030 China; ^3^ Department of Minimally Invasive Thoracic Surgery Center Second Affiliated Hospital of Naval Medical University Shanghai 201209 China; ^4^ Department of Medical Oncology Shanghai Pulmonary Hospital and Thoracic Cancer Institute, Tongji University School of Medicine Shanghai 200433 China; ^5^ Department of Plastic Surgery Renji Hospital, Shanghai Jiaotong University School of Medicine Shanghai 200001 China; ^6^ Department of Pharmaceutical Science College of Pharmacy and Health Science St John's University Queens NY 11439 USA

**Keywords:** dual‐pedicle flap, epithelialization, long‐segment tracheal restoration, tissue‐engineered trachea, vascularization

## Abstract

Long‐segment tracheal reconstruction remains a formidable clinical challenge due to failures of conventional tissue‐engineered trachea (TET) grafts, stemming from inadequate vascularization, compromised cartilaginous framework, and epithelial deficiency. Transcriptomic profiling of conventional TETs revealed enriched hypoxia and inflammatory pathways, underscoring the need for robust vascular and epithelial strategies. Herein, a dual‐pedicle biomimetic TET is developed to provide a stable cartilaginous framework for airway support, immediate blood supply for tissue perfusion, and functional epithelial lining for barrier protection. Autologous rabbit auricular skin is sutured into tubes for epithelial lining, while chondrocytes in photo‐crosslinkable decellularized Wharton's jelly matrix hydrogels formed C‐shaped constructs assembled into cartilage tubes. Both are pre‐vascularized separately in cervical muscle for 4 weeks, then assembled with preserved unilateral pedicles, yielding integrated TETs with dual‐pedicle vascular networks and enhanced biomechanical properties. Orthotopic tracheal transplantation of this construct into rabbits demonstrated sustained patency, minimal stenosis, and improved 28 day survival (60% vs 40% for single‐pedicle controls). Immunofluorescence confirmed reduced bacterial infiltration, apoptosis, and inflammation (TLR4, MPO), with restored epithelial barriers (Claudin‐1, β‐defensin 1) and airway differentiation (cytokeratin 8 upregulation). Transcriptomics validated regenerative pathways, including oxidative phosphorylation and tight junctions. This dual‐pedicle approach preempts pathological cascades, offering a promising paradigm for clinical tracheal regeneration.

## Graphical abstract

Overcoming the limitations of conventional grafts, a biomimetic trachea with two independent vascular pedicles enables rapid integration and sustained patency. This approach markedly improves survival rates, restores the epithelial barrier, and promotes regenerative pathways, presenting a transformative paradigm for clinical application.

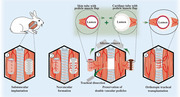

## 1 Introduction

The management of long‐segment tracheal defects, often resulting from extensive stenosis, tracheomalacia, or tumor resection, presents a formidable clinical challenge that can rapidly become life‐threatening.^[^ ^1^ ^]^ While end‐to‐end anastomosis is effective for short‐segment defects, this approach is doomed to fail when the resected portion exceeds 6 cm in children or 5 cm in adults, due to excessive anastomotic tension.^[^ ^2^ ^]^ The trachea's unique anatomical complexity and physiological demands have rendered conventional reconstruction methods using autologous tissues, allogeneic transplants, or synthetic prosthetics largely inadequate.^[^ ^3^ ^]^ Consequently, tissue engineering has emerged as the most promising frontier, offering the potential to construct viable, functional tracheal substitutes by integrating autologous cells with biocompatible scaffolds. Compared with traditional approaches, tissue engineering holds distinct advantages: it enables the design of scaffolds that replicate the mechanical strength of native cartilage, provides opportunities to pre‐vascularize constructs for improved graft survival, and supports re‐epithelialization essential for airway function, thereby holding immense promise for clinical translation.^[^ ^4^ ^]^

However, the clinical translation of tissue‐engineered tracheas (TETs) has been fraught with setbacks, tempering initial optimism. The pioneering work by Macchiarini et al., involving the transplantation of a decellularized allograft seeded with autologous cells, initially garnered global attention as a breakthrough.^[^ ^5^, ^6^, ^7^ ^]^ Subsequent long‐term follow‐up, however, revealed a starkly different reality. The patient suffered from graft malacia necessitating stent placement just 3 weeks post‐surgery, endured recurrent airway obstructions and infections.^[^ ^8^ ^]^ Scrutiny of this and other related studies exposed significant limitations, including overstated therapeutic effects and insufficient long‐term stability.^[^ ^9^, ^10^, ^11^ ^]^ Similar challenges were encountered by Xu et al., whose use of a stem‐cell‐seeded acellular dermal matrix also led to complications requiring stent placement in most patients, yielding less than ideal outcomes.^[^ ^12^ ^]^ These high‐profile cases underscore a critical lesson: a simple cell‐seeded scaffold is insufficient. True, prior investigations have repeatedly demonstrated long‐lasting success hinges on simultaneously addressing three fundamental pillars: a stable, biomimetic cartilaginous framework; a robust and immediate blood supply; and a complete, functional epithelial lining.^[^ ^13^, ^14^ ^]^

Progress has been made on the first pillar—the cartilaginous framework. The field has evolved from constructing simple, integrated cartilage tubes,^[^ ^15^, ^16^ ^]^ to developing more biomimetic structures,^[^ ^17^, ^18^, ^19^ ^]^ culminating recently in the successful regeneration of highly biomimetic C‐shaped cartilage rings.^[^ ^20^ ^]^ This advancement, driven by a deeper understanding of native tracheal anatomy, has laid a solid foundation for providing the necessary mechanical support. Importantly, the C‐shaped cartilage framework not only maintains airway patency but also preserves compliance, while its open posterior region facilitates vascular ingrowth. However, a stable C‐shaped cartilaginous framework alone is insufficient for long‐term survival in vivo.^[^ ^21^ ^]^

Of the remaining pillars, establishing a rapid and stable blood supply remains the most formidable challenge and a critical bottleneck for clinical success. Insufficient vascularization is the primary cause of graft necrosis, stenosis, and ultimate failure.^[^ ^22^ ^]^ Existing strategies have proven suboptimal. Forearm flap prefabrication followed by microvascular anastomosis is technically demanding and provides inadequate perfusion for longer grafts.^[^ ^23^, ^24^, ^25^ ^]^ Omentum wrapping, while effective, is highly invasive and associated with significant morbidity. Simpler methods, such as direct intramuscular or subcutaneous implantation, suffer from low efficiency, as neovessels struggle to penetrate the core of larger TET constructs, leaving the central region ischemic.^[^ ^26^, ^27^, ^28^, ^29^, ^30^ ^]^ Clearly, a novel, more efficient vascularization strategy is not just beneficial, but absolutely necessary for the survival of large‐volume TET grafts.

Finally, achieving rapid re‐epithelialization is crucial for preventing bacterial infection, inhibiting granulation tissue formation, and restoring airway barrier function.^[^ ^31^ ^]^ While seeding epithelial cells or transplanting cell sheets has shown some promise,^[^ ^32^, ^33^, ^34^ ^]^ these methods often fail to form an immediate, tight physical barrier. Our previous work identified autologous skin as a readily available and robust source for generating a protective squamous epithelial lining that effectively inhibits granulation tissue.^[^ ^35^ ^]^ However, integrating this epithelial component into a pre‐vascularized, load‐bearing biomimetic TET within a single, coherent strategy remains an unaddressed challenge.

**Scheme 1:**
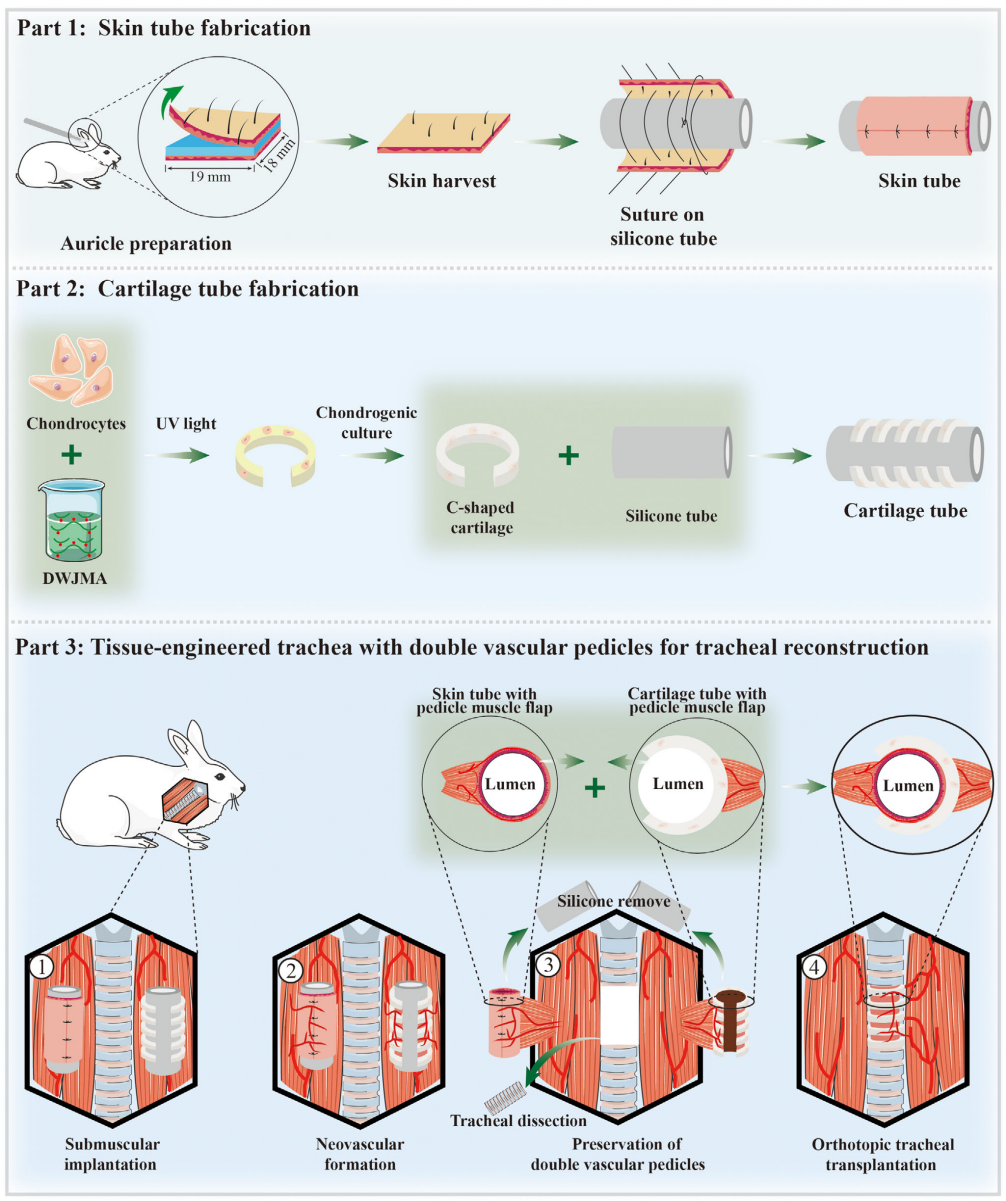
Fabrication of a Biomimetic TET with Dual Vascular Pedicles for Long‐Segment Tracheal Reconstruction. The process commences with harvesting auricular skin and cartilage from a rabbit. The auricular skin is carefully dissected and sutured around a silicone tube to form a skin tube, serving as the epithelial lining. Simultaneously, chondrocytes are isolated from the auricular cartilage of the same rabbit and uniformly suspended in a photo‐crosslinked DWJMA hydrogel. This cell‐hydrogel mixture is cultured in vitro under chondrogenic conditions to produce C‐shaped cartilage constructs. Multiple C‐shaped cartilages are then assembled around a silicone tube to create a cartilage tube. For in vivo maturation, both the skin and cartilage tubes are implanted separately into the cervical muscles on either side of the trachea in an autologous rabbit model. After 4 weeks, both constructs achieve robust vascularization, during which the C‐shaped cartilages in the cartilage tube mature and fuse into a stable, integrated structure. Subsequently, the vascularized skin and cartilage tubes are dissected from surrounding tissues, each retaining a unilateral pedicled muscle flap to maintain blood supply. Following removal of the inner silicone tubes, the vascularized skin tube is nested within the matured cartilage tube, yielding an integrated TET that biomimetically recapitulates the native trachea's essential features: a C‐shaped cartilaginous framework, functional epithelial lining, and dual‐pedicle vascular network. This highly vascularized, epithelium‐lined biomimetic TET is then employed to repair a long‐segment tracheal defect in the autologous rabbit model, highlighting its potential for clinical translation.

Therefore, this study proposes an innovative and integrated solution designed to simultaneously address all three critical pillars of tracheal reconstruction. We hypothesize that by prefabricating two separate, vascularized components in vivo—a biomimetic C‐shaped cartilage tube and an autologous skin tube—and then assembling them while preserving their individual vascular pedicles, we can create a highly biomimetic TET with a novel dual‐pedicle blood supply. This strategy, outlined in **Scheme** **1**, is engineered to provide robust, immediate, and comprehensive vascularization to the entire composite TET graft. We will evaluate the efficacy of this dual‐pedicle approach and assess the protective function of the integrated squamous epithelium following in situ repair of a long‐segment tracheal defect in a rabbit model. This work aims to establish a new paradigm for constructing a fully viable and functional TET, paving the way for future clinical applications.

## 2 Results

### 2.1 Conventional TET Grafts Display a Transcriptomic Profile of Severe Hypoxia and Uncontrolled Inflammation

**Figure 1:**
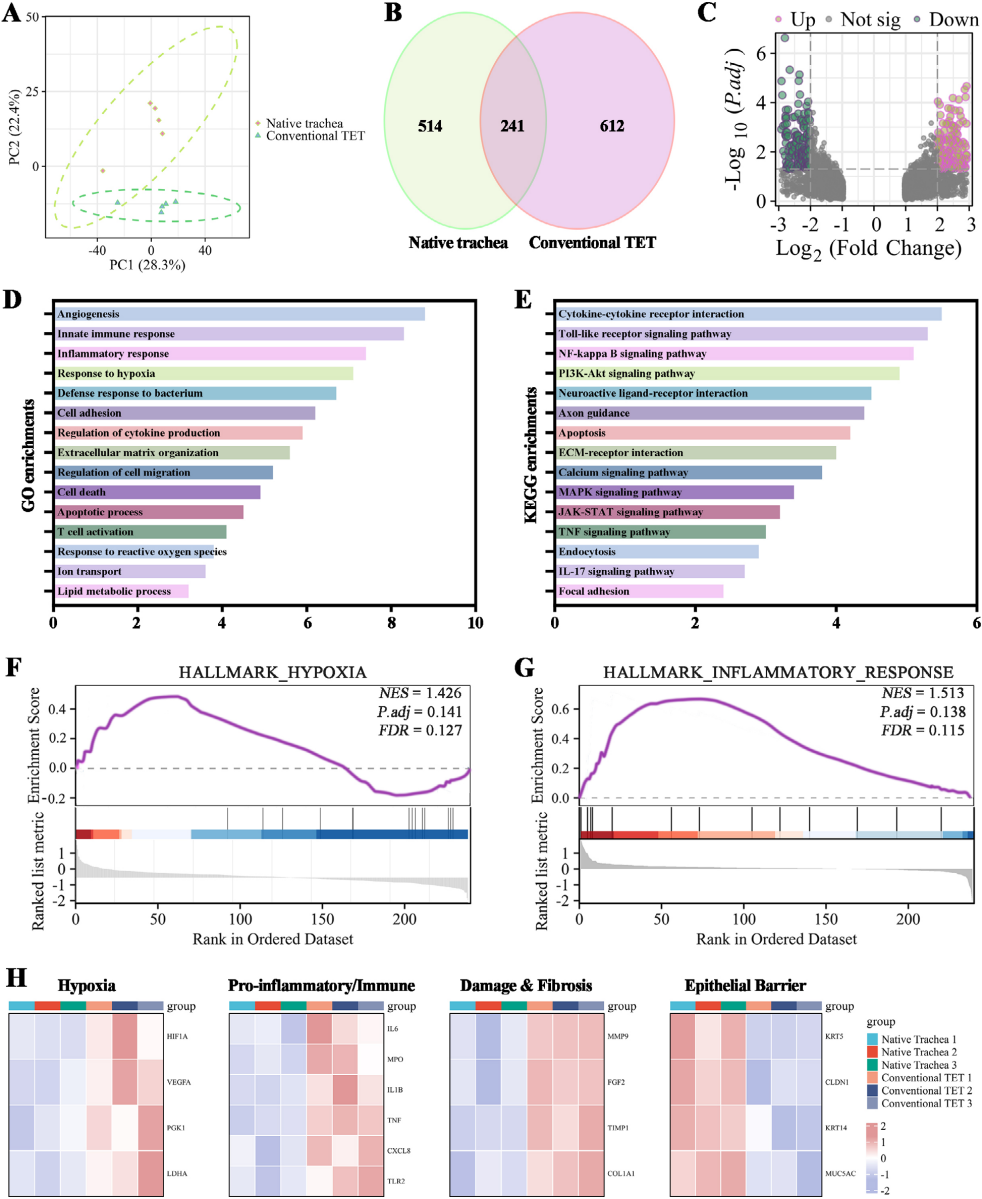
Transcriptomic profiling reveals failure of conventional TET grafts due to severe hypoxia and uncontrolled inflammation. A) PCA showing distinct separation of global transcriptomic profiles between conventional TET grafts and native trachea controls. B) Heatmap and C) volcano plot visualizing DEGs between the two groups, with significant DEGs defined by a fold change > 2 and adjusted *p*‐value <0.05. D) GO and E) KEGG pathway analyses of upregulated genes in TET grafts, indicating strong enrichment for pathways related to hypoxia and inflammatory responses. GSEA confirming significant positive enrichment for the “Hallmark Hypoxia” F) and “Hallmark Inflammatory Response” G) gene sets in the conventional TET group. H) Heatmap of core enrichment genes from GSEA, highlighting upregulation of genes associated with hypoxia, pro‐inflammatory/immune signaling, tissue damage, and fibrosis, alongside downregulation of genes linked to a healthy epithelial barrier.

To elucidate the molecular basis of conventional TET graft failure, we performed RNA sequencing on conventional TET grafts at 4 weeks post‐transplantation and compared them to healthy native tracheas (*n* = 3 per group). Principal Component Analysis (PCA) revealed distinct transcriptomic profiles, with conventional TETs forming a separate cluster from native tracheas, indicating significant molecular dysregulation (**Figure** **1**A). Differential expression analysis identified 1126 differentially expressed genes (DEGs), with 514 downregulated and 612 upregulated in conventional TETs compared to native trachea (Figure 1B,C). Gene Ontology (GO) and Kyoto Encyclopedia of Genes and Genomes (KEGG) pathway analyses of upregulated DEGs showed strong enrichment for hypoxia‐related (“Response to Hypoxia”) and inflammatory pathways (“Inflammatory Response,” “TNF Signaling Pathway”) (Figure 1D,E; Figure S1, Supporting Information). Gene Set Enrichment Analysis (GSEA) confirmed significant positive enrichment for “Hallmark Hypoxia” and “Hallmark Inflammatory Response” gene sets in conventional TETs (Figure 1F,G). A heatmap of core enrichment genes highlighted upregulated hypoxia (HIF1A, VEGFA), inflammation (IL6, TNF), tissue damage (MMP9), and fibrosis (FGF2) genes, alongside downregulated epithelial barrier genes (CLDN1, KRT5) (Figure 1H). These findings indicate that conventional TET failure is driven by a pathological molecular environment dominated by hypoxia and inflammation.

### 2.2 Muscle Encapsulation Enhances Vascularization of Autologous Skin Tube

**Figure 2:**
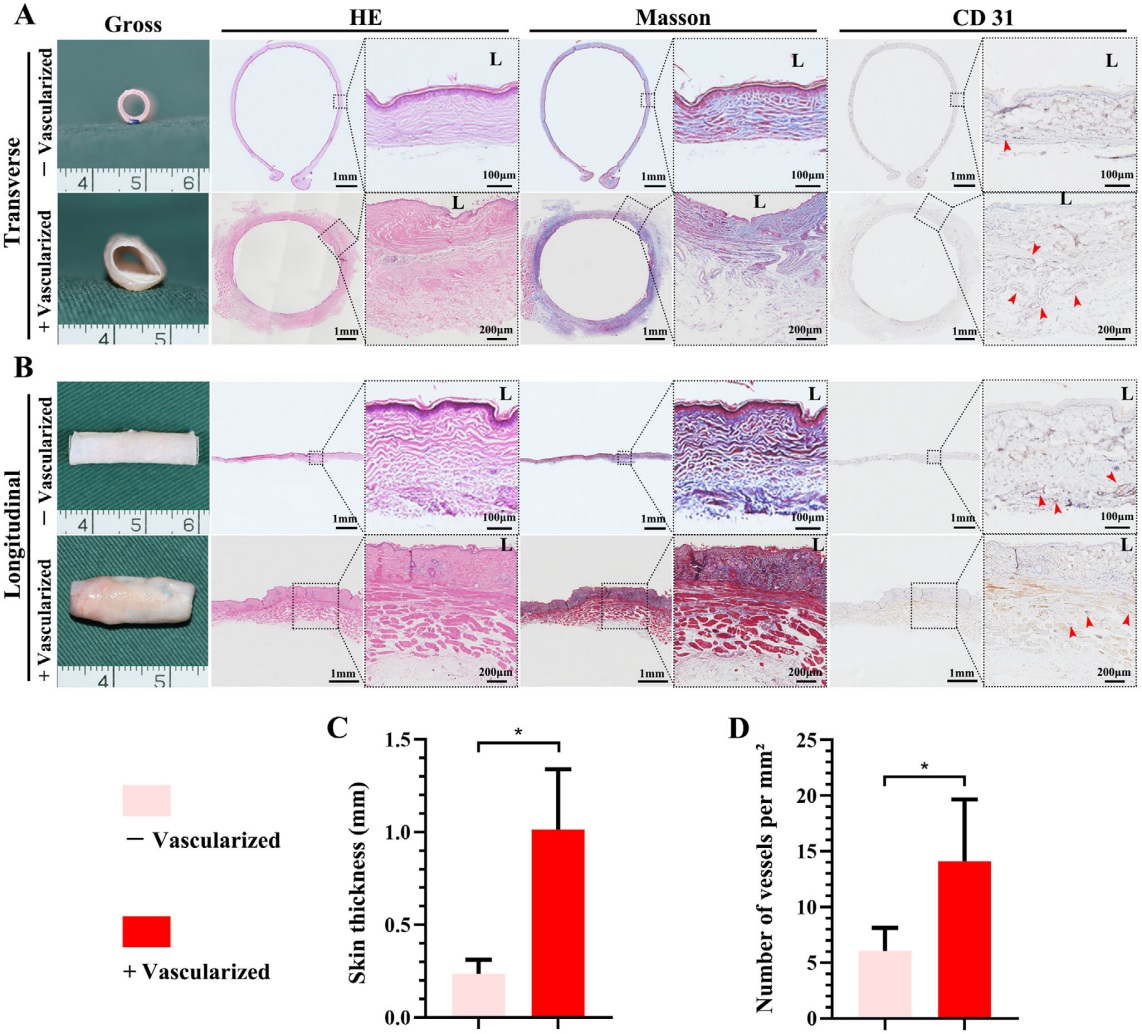
In Vivo Maturation Yields a Structurally Stable and Highly Vascularized Epithelial Module. Characterization of the engineered skin tube before (pre‐implantation, ‐vascularization) and after 4 weeks of in vivo maturation in the cervical muscle (+vascularization). Representative images of transverse A) and longitudinal B) sections showing gross morphology, tissue structure (HE), and collagen deposition (Masson's Trichrome). Immunohistochemistry for the endothelial marker CD31 (red arrows) reveals marked neovessel formation throughout the flap. Quantitative analyses indicating significant increases in skin flap thickness C) and microvessel density (D) in the +vascularization group. Data are presented as mean ± SD (*n* = 5 per group, ^*^ *p* < 0.05). “L” denotes the luminal side of the tube.

To address hypoxia and inflammation in TET grafts, we investigated muscle encapsulation to promote vascularization of the skin tube. After 4 weeks of implantation in the rabbit cervical muscle, the skin tube exhibited increased thickness and a color shift from pale to light red, indicating vascular integration (**Figure** **2**A,B). Histologically, hematoxylin‐eosin (HE) staining revealed that gaps in the sutured skin were bridged by fibrous tissue, forming a sealed tubular structure. Masson's trichrome staining confirmed the addition of fibrous connective and muscle tissue on the outer layer, contributing to increased wall thickness. CD31 immunohistochemistry showed a significant increase in microvessel density in the outer fibrous and muscular layers post‐vascularization (Figure 2A,B). Quantitative analysis confirmed a significant increase in skin flap thickness (Figure 2C) and microvessel density (Figure 2D), demonstrating that muscle encapsulation promotes robust vascularization and structural integrity of the skin tube.

### 2.3 In Vitro and In Vivo Maturation of C‐Shaped Cartilage Constructs

To mimic the native tracheal cartilage, we developed C‐shaped constructs using a photo‐crosslinkable decellularized Wharton's jelly matrix (DWJMA) hydrogel and a custom C‐shaped mold (inner diameter 6 mm, outer diameter 8 mm, height 1.8 mm, 75% circumferential) (Figure S2A, Supporting Information). Photo‐crosslinking produced stable, acellular C‐shaped hydrogels that accurately replicated the mold's geometry (Figure S2B, Supporting Information). For the generation of the final cartilage module, chondrocytes were homogeneously suspended within the hydrogel precursor prior to casting (1 × 10^8^ cells mL^−1^). This resulted in a cell‐laden construct with uniformly distributed chondrocytes, ready for subsequent in vitro or in vivo culture and maturation (Figure S2C, Supporting Information).

Chondrocyte‐laden hydrogels were cultured in vitro for 4 or 8 weeks, showing progressive cartilage maturation (Figure S3A,B, Supporting Information). At 8 weeks, HE, Safranin‐O, Masson's trichrome, and type II collagen immunohistochemistry revealed denser tissue and increased glycosaminoglycan (GAG) and collagen II deposition compared to 4 weeks. Biochemical analysis confirmed significant increases in DNA, GAG, and collagen II content from 4 to 8 weeks (Figure S3C, Supporting Information), with corresponding improvements in compressive and fracture strength (Figure S3D, Supporting Information). However, these properties remained inferior to native tracheal cartilage, indicating limitations of in vitro maturation for clinical‐grade cartilage.

To overcome in vitro limitations, chondrocyte‐laden C‐shaped hydrogels were implanted subcutaneously in nude mice for 4 or 8 weeks. Constructs retained their C‐shaped morphology (Figure S4A,C, Supporting Information) and exhibited progressive maturation, with histological analysis showing increased tissue density, cellular organization, and cartilage‐specific extracellular matrix (ECM) deposition (HE, Safranin‐O, type II collagen) by 8 weeks (Figure S4B,D, Supporting Information). Biochemical analysis revealed significant increases in GAG and total collagen contents, approaching native tracheal cartilage levels at 8 weeks (Figure S4E, Supporting Information). Biomechanical testing showed enhanced compressive strength (horizontal and vertical orientations) and fracture resistance, achieving equivalence to native tracheal cartilage by 8 weeks (Figure S4F, Supporting Information). These results demonstrate that in vivo maturation produces biochemically and biomechanically competent cartilage, surpassing in vitro outcomes.

### 2.4 Vascularized Cartilage Tube Development in Rabbits

**Figure 3:**
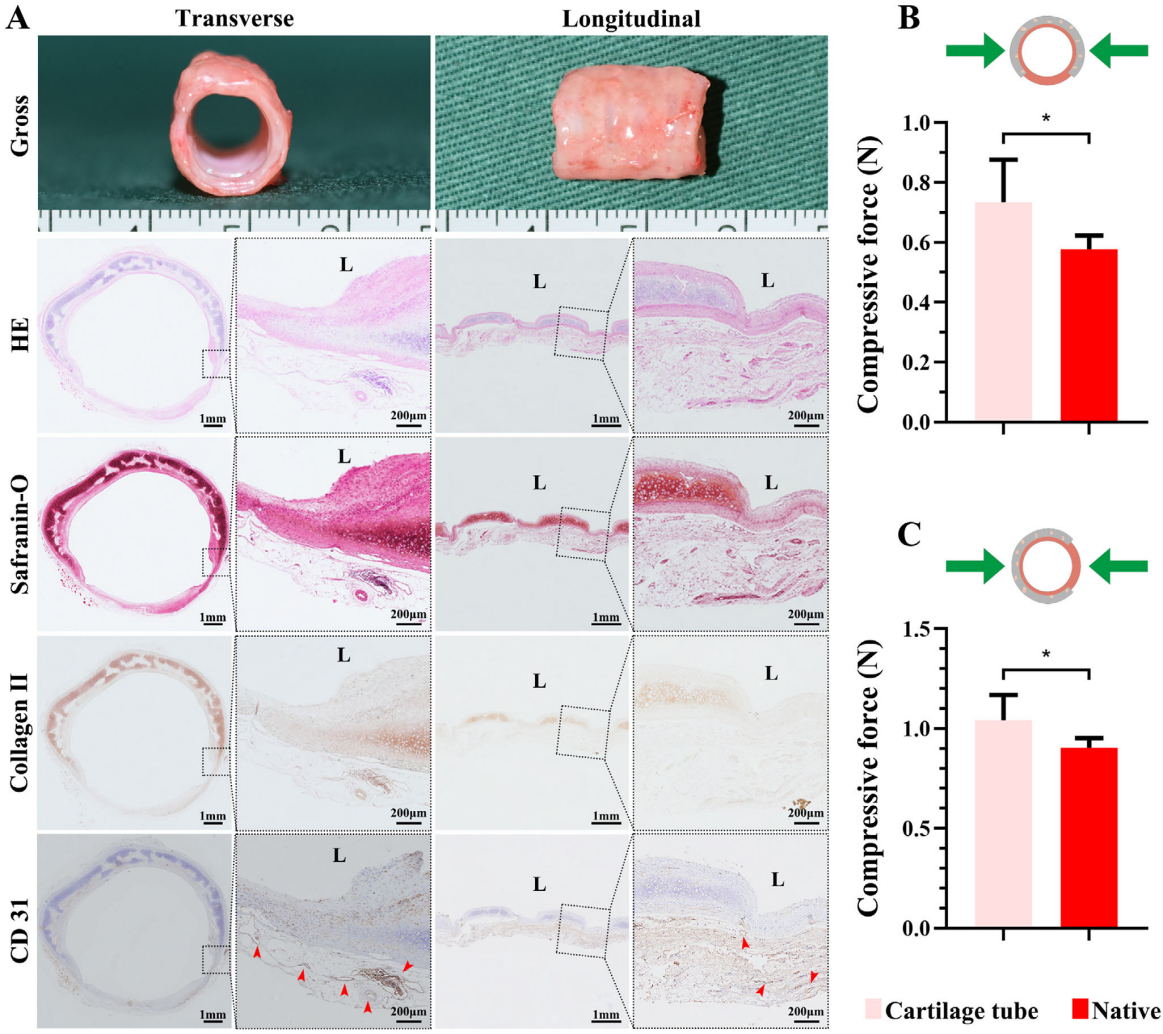
In Vivo Engineered Cartilage Tube Exhibits Mature and Vascularized Structure. A) Characterization of the cartilage tube following 4 weeks of in vivo maturation in a rabbit model. Histological analysis (HE) reveals a stable, integrated cartilaginous structure. Safranin‐O staining and immunohistochemistry for type II collagen confirm robust deposition of cartilage‐specific ECM, rich in GAGs and type II collagen, respectively. Immunohistochemistry for CD31 (red arrows) demonstrates extensive neovascularization throughout the cartilage tube. Biomechanical testing indicates that the engineered cartilage tube exhibits stress–strain profiles superior to those of native trachea under lateral B) and anterior–posterior C) compressive forces, confirming its mechanical suitability for tracheal replacement. Data are presented as mean ± SD (*n* = 5 per group, ^*^ *p* < 0.05). “L” denotes the luminal side of the tube.

C‐shaped chondrocyte‐loaded hydrogels were assembled around a silicone tube and implanted into the rabbit cervical muscle for 4 weeks. The resulting cartilage tube displayed a red–pink appearance, resembling native trachea, with visible cartilage rings (**Figure** **3**A). HE staining showed cartilage‐like structure in cross‐sections and fibrous tissue connecting rings in longitudinal sections. Safranin‐O and type II collagen staining confirmed robust cartilage ECM. CD31 immunohistochemistry revealed significant vascularization in the outer connective tissue but minimal intraluminal vascular ingrowth due to the silicone stent (Figure 3A). Biomechanical testing indicated superior compressive strength in lateral and anterior–posterior directions compared to native trachea (Figure 3B,C). Biochemical analysis showed elevated GAG and collagen II levels relative to native trachea (Figure S5A,B, Supporting Information), confirming effective vascularization and cartilage maturation via muscle encapsulation.

### 2.5 Dual‐Pedicle TET Mimics Native Trachea Structure

**Figure 4:**
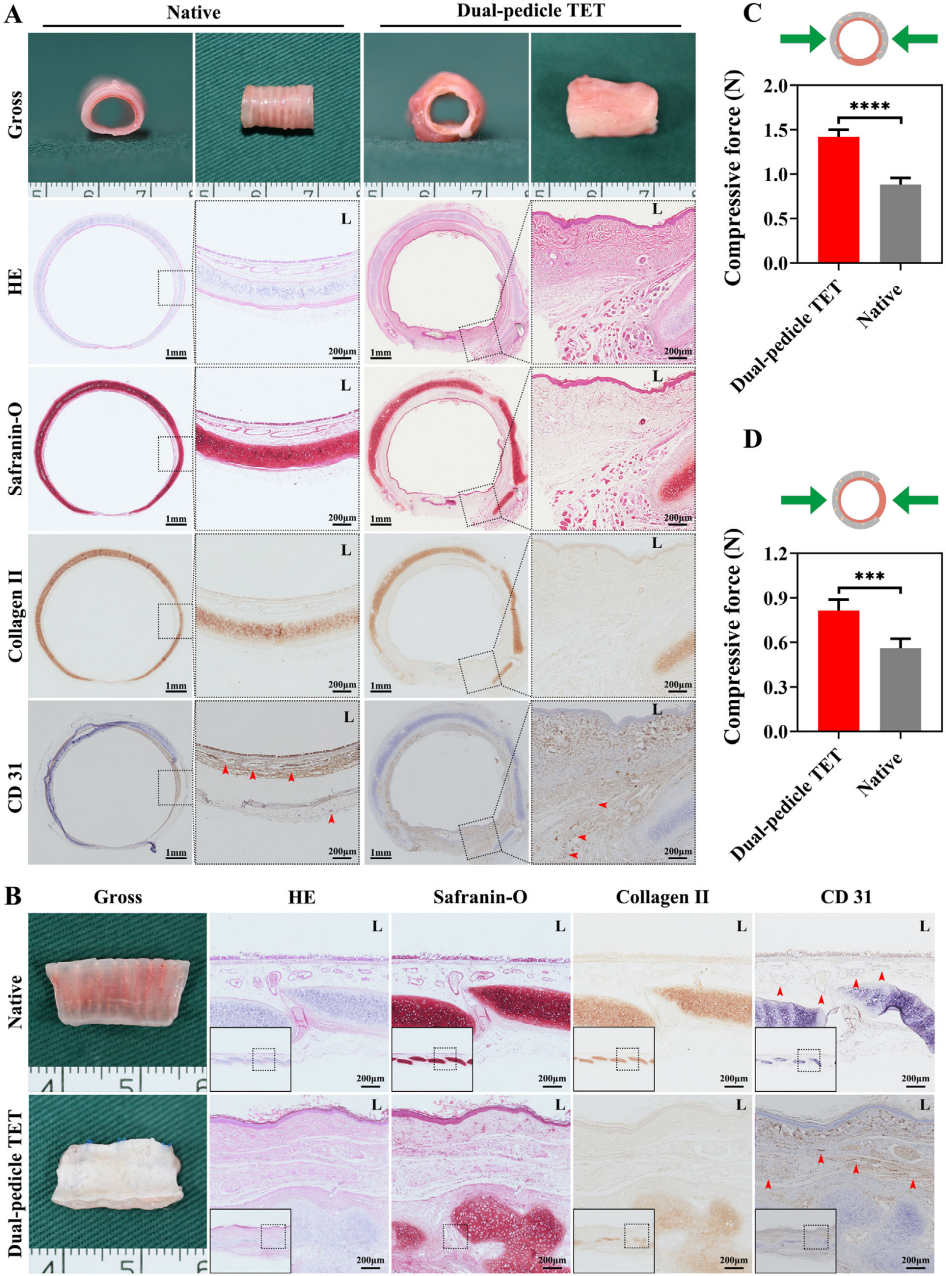
Assembled Dual‐Pedicle TET Closely Mimics the Structural and Mechanical Properties of Native Trachea. Characterization of the assembled dual‐pedicle TET, demonstrating successful integration of pre‐vascularized and pre‐epithelialized modules. Gross morphology and histological analysis of transverse A) and longitudinal B) sections confirm a biomimetic structure, comprising a continuous inner epithelial lining (HE staining) and a stable outer cartilaginous framework (Safranin‐O and immunohistochemistry collagen II stainings). Immunohistochemistry CD31 staining (red arrows) reveals a well‐distributed vascular network throughout both epithelial and cartilaginous components. Biomechanical assessment under lateral C) and anterior–posterior D) compression shows that the stress–strain profile of the dual‐pedicle TET surpasses that of native trachea. Data are presented as mean ± SD (*n* = 5 per group, ^***^ *p* < 0.001, ^****^ *p* < 0.0001). “L” denotes the luminal side of the tube.

The dual‐pedicle TET, integrating vascularized skin and cartilage tubes, exhibited a patent lumen and smooth epithelial lining (Figure 4A,B). HE staining revealed a biomimetic structure with an inner epithelial layer, outer C‐shaped cartilage, and vascularized connective tissue at cartilage gaps. Safranin‐O and type II collagen staining confirmed uniform cartilage ECM, resembling native trachea. CD31 immunohistochemistry showed extensive vascularization between cartilage and epithelium, comparable to native trachea (Figure 4A,B). Biomechanical testing demonstrated superior compressive strength in lateral and anterior–posterior directions compared to single‐pedicle TETs and native trachea (Figure 4C,D). Biochemical analysis indicated GAG and collagen II levels comparable to native trachea (Figure S6A,B, Supporting Information). Immunofluorescence staining for pan‐cytokeratin and E‐cadherin confirmed a robust epithelial lining in dual‐pedicle TETs, absent in cartilage tube‐only constructs (Figure S7, Supporting Information), underscoring the necessity of the skin tube for epithelial formation.

### 2.6 Dual‐Pedicle TET Enhances Tracheal Defect Repair and Survival

**Figure 5:**
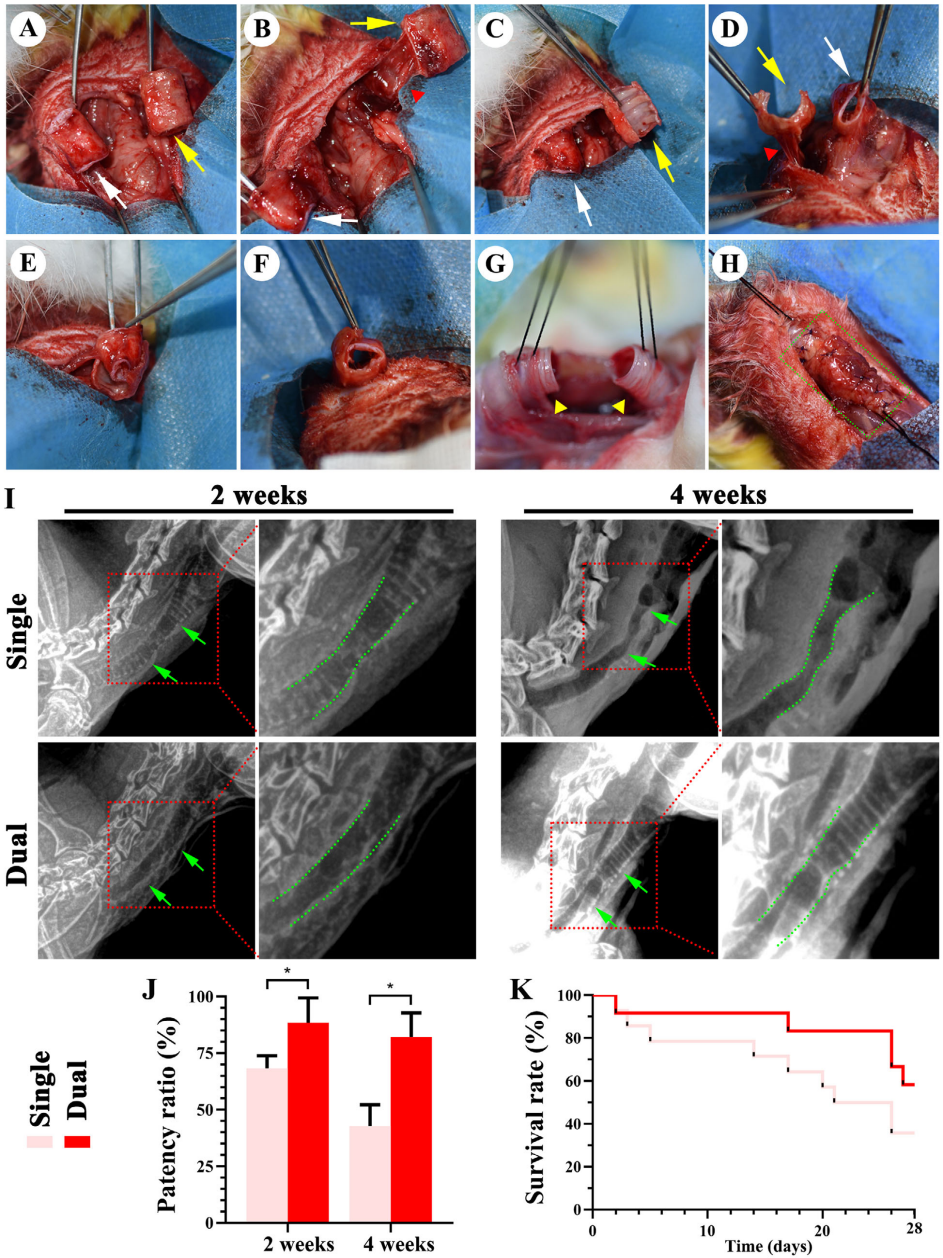
Dual‐Pedicle Strategy Enhances Orthotopic Tracheal Reconstruction and Improves Postoperative Survival. Illustration of the surgical procedure for orthotopic tracheal transplantation in a rabbit model. A, B) Exposure of the pre‐vascularized skin (white arrows) and cartilage (yellow arrows) modules, preserving their distinct vascular pedicles (red triangles). C–E) The skin tube is nested within the cartilage tube and assembled in vivo. F) The construct is stented and allowed to integrate for 2 weeks. G) A long‐segment defect is created in the native trachea (yellow arrowheads). H) The fully assembled dual‐pedicle TET is anastomosed end‐to‐end to bridge the defect. I) Radiographic imaging at 2 and 4 weeks reveals sustained airway patency (green dashed line) in the dual‐pedicle group, while the single‐pedicle control group exhibits progressive stenosis. J) Quantitative analysis of the tracheal patency ratio confirms these findings. K) The dual‐pedicle TET group demonstrates a significantly higher 28 day survival rate. Data are presented as mean ± SD (*n* = 10 per group, ^*^ *p* < 0.05).

**Figure 6:**
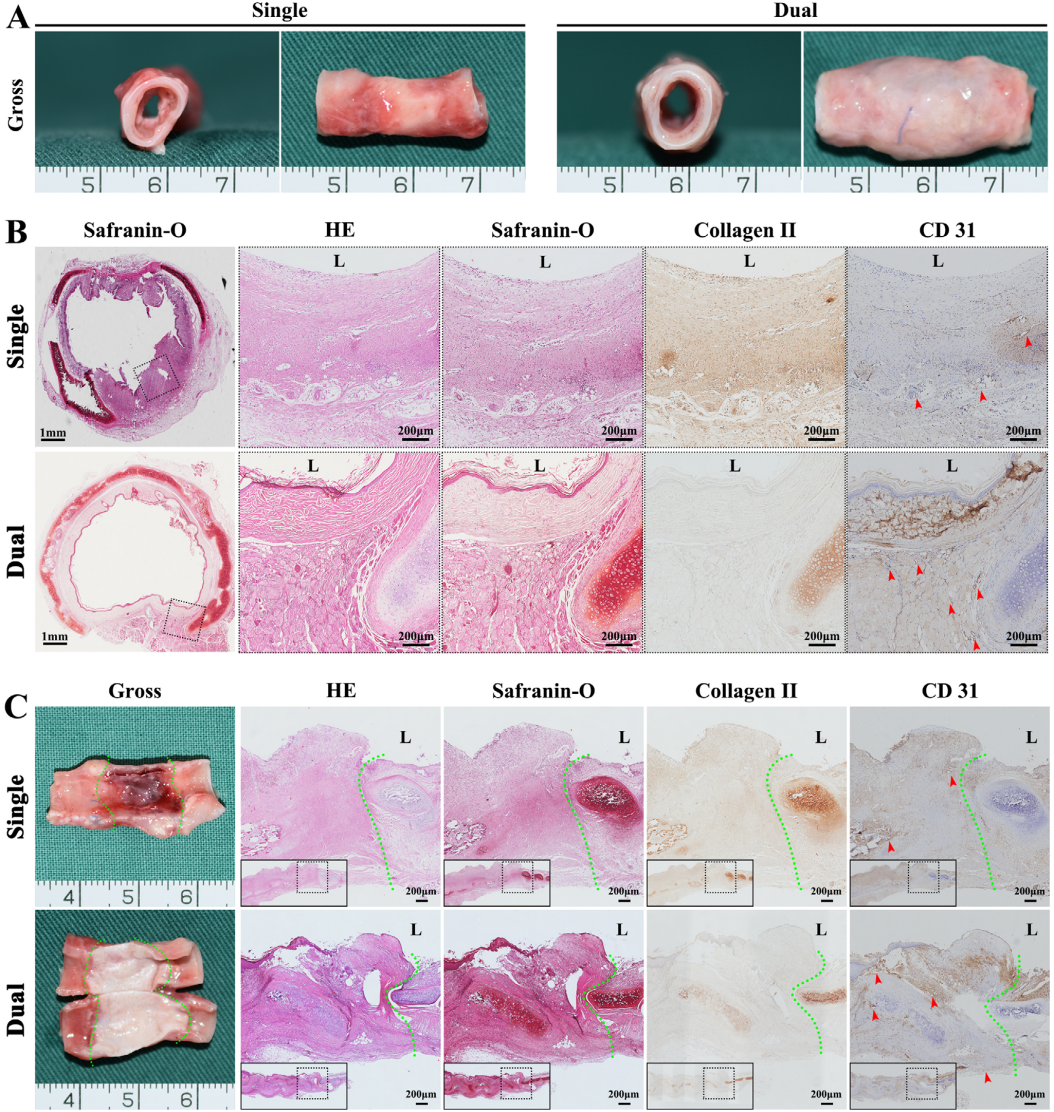
Dual‐Pedicle Strategy Ensures TET Graft Viability, Vessel Perfusion, and Cartilage Maintenance. Gross and histological assessments at 4 weeks post‐transplantation highlight distinct outcomes for single‐pedicle and dual‐pedicle TET groups. Cross‐sectional A,B) and longitudinal C) views of dual‐pedicle TETs demonstrate seamless integration with the host trachea (anastomosis at green dotted line) and maintenance of a stable, open lumen. Histological analysis (HE, Safranin‐O, type II collagen) confirms a healthy, continuous epithelial lining, a mature cartilaginous framework, and robust submucosal vascularization (CD31, red arrows). In contrast, single‐pedicle TETs exhibit severe chondromalacia, with collapse of the cartilaginous support structure, loss of matrix integrity, and epithelial sloughing. “L” denotes the luminal side of the tube.

**Figure 7:**
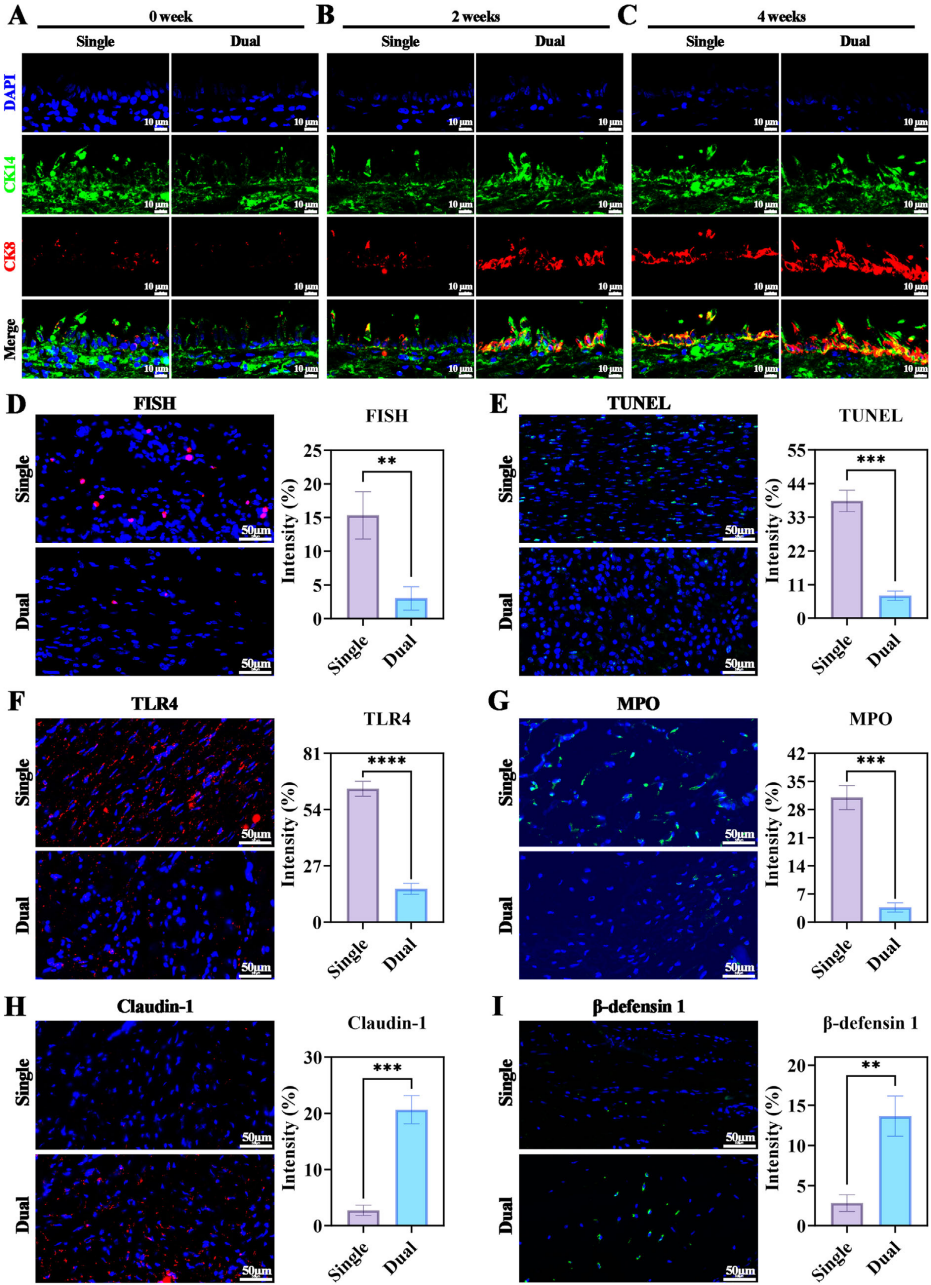
Dual‐Pedicle Design Reduces Bacterial Infiltration, Cell Death, and Inflammation while Promoting Epithelial Barrier Restoration. Immunofluorescence staining for skin epithelium‐specific marker CK14 and airway epithelium‐specific marker CK8 in the single‐pedicle TET and dual‐pedicle TET groups at 0, 2, and 4 weeks A–C). Single‐pedicle grafts exhibit severe bacterial infiltration D, FISH), widespread apoptosis E, TUNEL), and extensive inflammatory infiltrate, as indicated by elevated expression of the inflammatory receptor TLR4 F) and neutrophil marker MPO G). This pathological environment corresponds with a compromised epithelial barrier in the single‐pedicle group, evidenced by reduced expression of the tight junction protein Claudin‐1 H) and the antimicrobial peptide β‐defensin 1 (I). Quantitative analysis of each marker corroborates these observations. Data are presented as mean ± SD (*n* = 5 per group, ^*^ *p* < 0.05).

Dual‐pedicle TETs were used to repair 1.5 cm tracheal defects in rabbits, with single‐pedicle TETs as controls (**Figure** **5**A–H). X‐ray imaging at 2 weeks showed minimal stenosis in both groups, but by 4 weeks, single‐pedicle TETs exhibited significant stenosis, while dual‐pedicle TETs maintained stable patency (Figure 5I,J). Survival analysis revealed a 60% survival rate at 4 weeks for the dual‐pedicle group compared to 40% for the single‐pedicle group (Figure 5K). Gross and histological images indicated that the single‐pedicle TETs displayed collapsed cartilage, irregular lumens, and reduced ECM (HE, Safranin‐O, type II collagen), while the dual‐pedicle TETs maintained intact cartilage, smooth lumens, and robust vascularization (CD31) (**Figure** **6**A–C). Biochemical and biomechanical analyses confirmed higher GAG and collagen II content and superior mechanical strength in dual‐pedicle TETs, resembling native trachea, whereas single‐pedicle grafts showed significant degradation (Figure S8A,B, Supporting Information). Immunofluorescence for CK14 (skin epithelium) and CK8 (airway epithelium) revealed progressive epithelial differentiation in both groups, with significantly higher CK8 intensity in dual‐pedicle TETs at 2 and 4 weeks, indicating enhanced airway epithelial transition (**Figure** **7**A–C; Figure S9, Supporting Information).

### 2.7 Dual‐Pedicle TET Mitigates Pathological Responses

Immunofluorescence analysis at 4 weeks post‐transplantation revealed severe pathological changes in single‐pedicle TETs, including bacterial infiltration (Fluorescence in situ hybridization, FISH), widespread apoptosis (Terminal deoxynucleotidyl transferase dUTP Nick End Labeling, TUNEL), and intense inflammation (Toll‐like receptor 4, TLR4; Myeloperoxidase, MPO) (Figure 7D–G). These grafts exhibited compromised epithelial barriers, with reduced Claudin‐1 and near‐absent β‐defensin 1 expression (Figure 7H,I). In contrast, dual‐pedicle TETs showed minimal bacterial infiltration, low apoptosis, and basal inflammatory marker levels, with robust, continuous Claudin‐1 and β‐defensin 1 expression, indicating a restored epithelial barrier (Figure 7D–I). Quantitative fluorescence intensity analysis confirmed these differences (*p* < 0.05).

### 2.8 Transcriptomic Analysis Reveals Molecular Basis of Dual‐Pedicle Success

**Figure 8:**
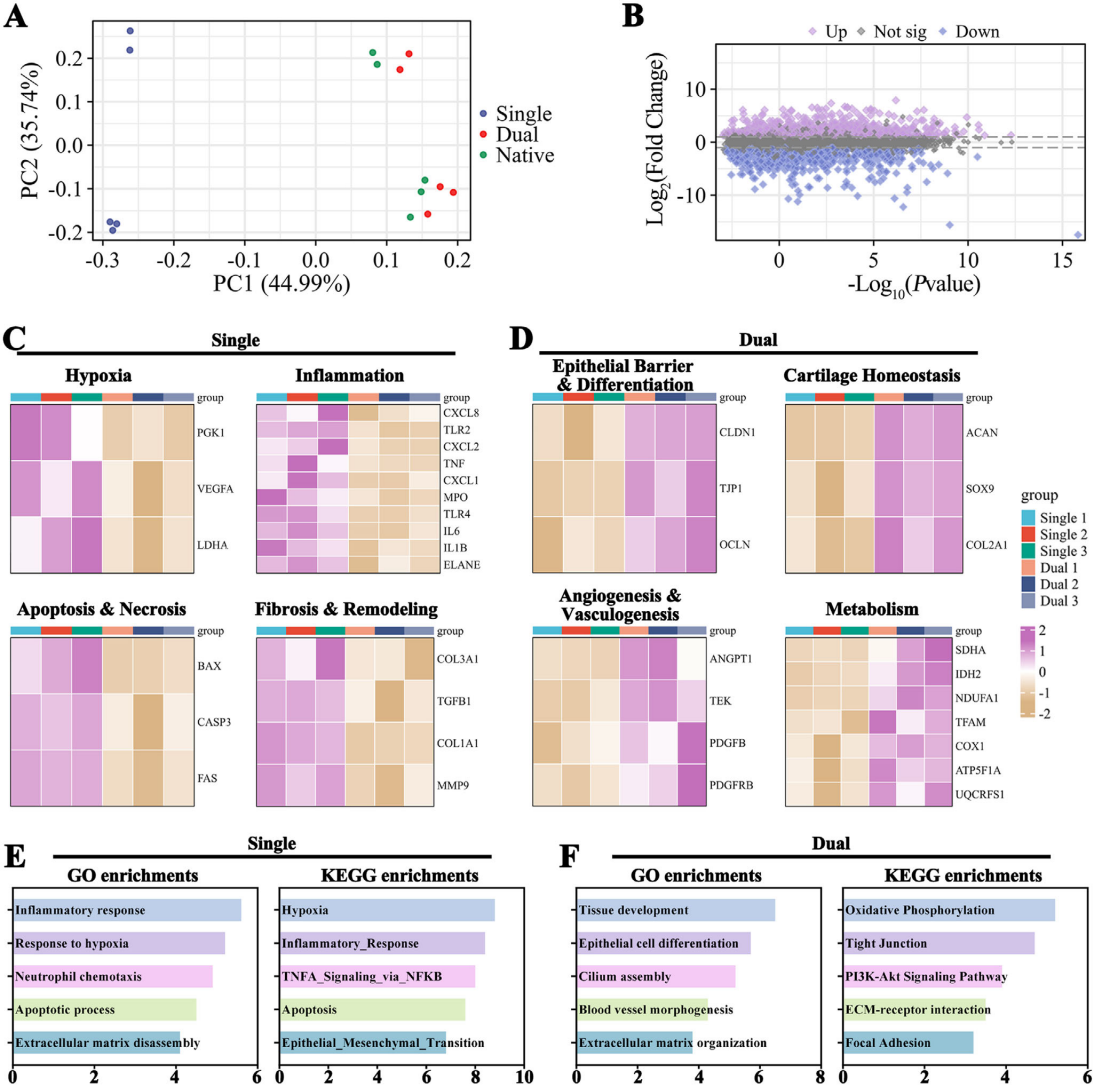
Transcriptomic Profiling Highlights Regenerative Success versus Pathological Failure. A) PCA reveals three distinct transcriptomic clusters, with the dual‐pedicle group clustering closely to native trachea, indicating successful molecular regeneration, while the single‐pedicle group is markedly separated, reflecting a pathological state. B) Volcano plot visualizing thousands of DEGs driving this divergence. Heatmap analysis illustrates opposing biological programs: single‐pedicle grafts exhibit upregulation of genes linked to hypoxia, inflammation, apoptosis, and fibrosis C), whereas dual‐pedicle grafts show robust expression of gene sets supporting epithelial barrier function, cartilage homeostasis, healthy angiogenesis, and efficient aerobic metabolism D). GO and KEGG analyses confirm distinct pathway enrichments: the single‐pedicle group is enriched for pathological pathways, including “Hallmark Hypoxia” and “Inflammatory Response” E), while the dual‐pedicle group is enriched for regenerative pathways, such as “Oxidative Phosphorylation" and “Tight Junction” F), quantitatively validating its restoration to a healthy, functional state.

RNA sequencing of single‐pedicle, dual‐pedicle, and native trachea tissues (*n* = 3 per group) showed distinct transcriptomic profiles (**Figure** **8**A). The dual‐pedicle group clustered closely with native trachea, while single‐pedicle TETs formed a distinct pathological cluster. Differential expression analysis identified thousands of DEGs (Figure 8B). Single‐pedicle TETs exhibited upregulated genes for hypoxia (HIF1A, PGK1), inflammation (IL1B, TNF, MPO), apoptosis (CASP3, BAX), and fibrosis (TGFB1, COL1A1) (Figure 8C; Figure S10A, Supporting Information). Conversely, dual‐pedicle TETs showed high expression of genes for epithelial barrier function (CLDN1, TJP1), cartilage homeostasis (SOX9, COL2A1), angiogenesis (ANGPT1, TEK), and aerobic metabolism (COX4I1, NDUFA9) (Figure 8D; Figure S10B, Supporting Information). Pathway analysis confirmed enrichment of pathological pathways (“Hypoxia,” “Inflammatory Response”) in single‐pedicle TETs and regenerative pathways (“Oxidative Phosphorylation,” “Tight Junction”) in dual‐pedicle TETs (Figure 8E,F). These data validate the dual‐pedicle strategy's role in promoting tracheal regeneration.

## 3 Discussion

Long‐segment tracheal reconstruction remains a formidable clinical challenge, with graft failure predominantly arising from inadequate cartilaginous framework, vascularization, and epithelialization.^[^ ^36^, ^37^, ^38^ ^]^ Vascularization serves as the cornerstone, as a robust blood supply not only sustains cartilage integrity by delivering oxygen and nutrients but also facilitates epithelialization by supporting cell migration, proliferation, and barrier formation.^[^ ^14^ ^]^ In turn, effective epithelialization is vital for maintaining an antibacterial and anti‐inflammatory microenvironment, preventing pathogen invasion, granulation tissue hyperplasia, and luminal stenosis.^[^ ^39^ ^]^ Insufficient vascularization triggers ischemia, leading to hypoxia‐driven cell death and inflammation, which compromises epithelial integrity and cartilage maintenance, perpetuating graft collapse.^[^ ^40^ ^]^ Our study elucidates these interdependencies at a molecular level, demonstrating that insufficient vascularization triggers a “hypoxia‐inflammation axis”, while dual‐pedicle pre‐vascularization preempts this cascade, enabling synergistic cartilage maintenance and epithelial restoration.

The vascularization achieved by conventional TET grafts with a single‐pedicle approach remained limited and insufficient to fully support the metabolic demands of the implanted construct.^[^ ^27^ ^]^ This limitation represents one of the core challenges that have so far prevented TET from being successfully applied in large‐animal models or translated into clinical practice.^[^ ^41^ ^]^ Specifically, although initial capillary ingrowth was observed, the vascular network was sparse and poorly organized, failing to provide adequate oxygen and nutrient delivery to deeper regions of the TET graft. More importantly, another fundamental difficulty for achieving functional long‐segment tracheal reconstruction is the rapid vascularization of the luminal surface of the TET, which is essential for prompt re‐epithelialization. Current pre‐vascularization strategies primarily promote vascularization around the cartilage component, but provide insufficient support for the luminal surface. To address this issue, the double‐pedicle vascularization strategy proposed in our study preserves bilateral muscle pedicle blood supply, thereby simultaneously supporting both cartilage and epithelial integration.

Transcriptomic profiling of conventional TET grafts revealed a pathological signature dominated by hypoxia (enriched “Hallmark Hypoxia” pathway, e.g., HIF1A, VEGFA) and inflammation (“Hallmark Inflammatory Response,” e.g., IL6, TNF), perpetuating a vicious cycle of matrix degradation (MMP9), apoptosis, and fibrosis (FGF2). This mirrors historical failures, such as Macchiarini's synthetic tracheal implants, which succumbed to ischemia‐induced necrosis and infection due to absent vascular networks, leading to high mortality in clinical trials.^[^ ^42^ ^]^ In contrast, our dual‐pedicle approach—modularly assembling separately vascularized skin and cartilage tubes—delivers immediate, robust blood supply to both structural and functional components, severing the hypoxia‐inflammation axis at its inception. Transcriptomic comparison between single‐ and dual‐pedicle TETs validates this: single‐pedicle grafts exhibit upregulated hypoxia (HIF1A, PGK1), inflammation (IL1B, TNF, MPO), apoptosis (CASP3, BAX), and fibrosis (TGFB1, COL1A1) pathways, leading to mechanical fragility and epithelial compromise. Dual‐pedicle TETs, however, mirror native trachea with enriched oxidative phosphorylation (indicating aerobic metabolism), tight junction (e.g., CLDN1, TJP1), and ECM‐receptor interaction pathways, supporting cartilage homeostasis (SOX9, COL2A1) and stable angiogenesis (ANGPT1, TEK). This aligns with recent advancements, such as the in situ vascularization of segmental tracheal scaffolds using poly(ε‐caprolactone) matrices, which enhanced endothelial integration and reduced hypoxia in preclinical models.^[^ ^43^ ^]^

The trachea's deceptively simple architecture—comprising C‐shaped cartilage for mechanical support, vascular networks for nutrient delivery, and pseudostratified ciliated epithelium for barrier function and mucociliary clearance—demands interdependent tissue engineering.^[^ ^16^, ^44^ ^]^ Building on our prior work with photo‐crosslinked DWJMA hydrogels, which enable tunable properties and biocompatibility for C‐shaped cartilage regeneration,^[^ ^20^ ^]^ we optimized maturation strategies. In vitro culture yielded progressive ECM deposition (GAG, collagen II) and biomechanical gains but fell short of native standards, highlighting nutrient diffusion limits. In vivo subcutaneous implantation in nude mice overcame this, achieving biomimetic biochemical and mechanical profiles by 8 weeks, likely due to enhanced paracrine signaling and vascular cues.^[^ ^45^ ^]^

For vascularization, cervical muscle encapsulation proved effective, yielding CD31‐positive vessels in outer connective tissues of both cartilage and skin tubes, consistent with prior reports.^[^ ^35^, ^46^ ^]^ CD31 immunohistochemistry was chosen to quantify microvessel density, as it specifically marks endothelial cells, providing direct evidence of neovascularization efficiency and its role in alleviating ischemia—critical for preventing the hypoxia seen in avascular grafts.^[^ ^47^ ^]^ However, intraluminal vessel penetration was limited by silicone stents, prompting our modular dual‐pedicle design. This ensured spatially comprehensive perfusion—outer for cartilage, inner for epithelium—resulting in superior biomechanical strength and ECM integrity compared to native trachea or single‐pedicle controls. Biomechanical testing (compressive and fracture strength) was essential to assess load‐bearing capacity under physiological stresses like coughing or ventilation, confirming the graft's mechanical suitability and highlighting vascularization's indirect support for cartilage resilience, as seen in partially decellularized scaffolds where preserved vascular cues enhanced neotissue regeneration.^[^ ^48^ ^]^ Although CD31 immunostaining confirmed increased vascular density in the dual‐pedicle group, our study did not include functional vascular imaging to directly visualize vascular connectivity and perfusion efficiency. Future investigations employing techniques such as micro‐CT angiography or indocyanine green fluorescence angiography will be essential to provide 3D and dynamic evidence of perfusion, thereby further strengthening the conclusions regarding the functional integration of the dual‐pedicle vascular network.

Epithelial reconstruction is equally critical, as delayed re‐epithelialization invites infection and stenosis.^[^ ^31^ ^]^ Leveraging autologous skin‐derived squamous epithelium, which we previously showed inhibits granulation,^[^ ^35^ ^]^ we formed vascularized skin tubes that integrated seamlessly, forming a submucosal‐like layer. Immunofluorescence for markers like pan‐cytokeratin (broad epithelial integrity) and E‐cadherin (cell adhesion) confirmed robust lining formation,^[^ ^49^, ^50^ ^]^ while Claudin‐1 (tight junctions) and β‐defensin 1 (antimicrobial defense) assessed barrier function and innate immunity—key to resisting luminal pathogens and inflammation,^[^ ^51^, ^52^ ^]^ deficits that exacerbate stenosis in epithelium‐deficient grafts.^[^ ^53^ ^]^ Notably, progressive CK8 upregulation (airway marker) over CK14 (skin marker) suggested metaplasia toward respiratory epithelium,^[^ ^54^, ^55^ ^]^ enhanced in dual‐pedicle TETs, aligning with studies on buccal mucosa transplantation where epithelial coverage prevents luminal obstruction.^[^ ^24^ ^]^ This is further supported by recent cell‐free tracheal scaffolds with sequential release of growth factors, which promoted epithelialization and vascularization for defect repair.^[^ ^56^ ^]^

Our findings further highlight that insufficient vascularization critically compromises the survival of epithelial grafts. In the non‐vascularized group, the epithelial lining failed to maintain long‐term viability, as evidenced by detachment, necrosis, and the absence of a continuous, differentiated epithelial layer on histological examination. These changes were accompanied by prominent inflammatory cell infiltration, suggesting that inadequate blood supply not only deprives the epithelium of oxygen and nutrients but also predisposes the graft to secondary inflammatory damage. Consistently, immunostaining for CK14 confirmed the loss of epithelial integrity in the non‐vascularized controls, whereas the double‐pedicle pre‐vascularization group preserved a continuous epithelial sheet with markedly improved survival and organization. These results underscore the necessity of establishing a robust and prompt vascular network to sustain epithelial graft viability. From a translational perspective, rapid and stable vascularization of the luminal surface is essential for successful tracheal regeneration, as it ensures early re‐epithelialization, prevents infection, and provides long‐term functional airway protection.

In rabbit orthotopic long‐segment tracheal reconstruction models, dual‐pedicle TETs outperformed single‐pedicle controls, maintaining patency (minimal stenosis at 4 weeks), reducing chondromalacia, and boosting 28 day survival. In the double‐pedicle group (*n* = 10), 28 day survival was 6/10. The four non‐survivors died from airway‐centric complications rather than catastrophic loss of pedicle perfusion, including early postoperative mucus plugging with edema‐related luminal narrowing, aspiration‐associated pneumonia leading to sepsis, and progressive anastomotic granulation causing critical stenosis. In the single‐pedicle group (*n* = 10), 28 day survival was 4/10. The six deaths were predominantly attributable to inadequate luminal support and poorer perfusion, presenting as early epithelial sloughing with necrotic debris obstruction, bacterial colonization/infection, severe anastomotic granulation with fixed stenosis, and (in isolated cases) segmental collapse consistent with tracheomalacia. These findings support that enhanced and durable vascularization reduces airway‐related complications and improves short‐term survival.

Although the double‐pedicle strategy significantly improved outcomes compared with the single‐pedicle approach, the 28 day survival rate of 60% indicates that further refinement is required. Mortality in this challenging rabbit model is likely multifactorial, involving postoperative airway management, anastomotic granulation and stenosis, infection, and the inherent fragility of the airway. These findings underscore the need for systematic optimization, including improved perioperative care, adjunctive measures such as temporary stenting to maintain luminal patency, and extending the pre‐vascularization period to stabilize the microvascular network. Continued refinement of these parameters is expected to further enhance survival and accelerate translation of this strategy toward clinical application.

Histological and immunofluorescence analyses corroborated the above conclusions: single‐pedicle grafts suffered bacterial infiltration (FISH‐positive, to detect microbial invasion directly), apoptosis (TUNEL, quantifying cell death as a hypoxia outcome), inflammation (TLR4, MPO, marking immune activation), and barrier defects (low Claudin‐1, β‐defensin 1), while dual‐pedicle TETs exhibited restored epithelial integrity and basal inflammation. X‐ray patency ratios and survival curves quantified functional outcomes, linking vascular‐epithelial synergy to clinical relevance, akin to 3D‐printed tubular flaps that improved vascularization in tracheal reconstruction.^[^ ^57^ ^]^

In our study, hypoxia‐ and inflammation‐related pathways were identified in the transcriptomic analysis of the traditional TET group. Although we did not perform additional in‐depth mechanistic studies specifically targeting inflammation, our immunofluorescence findings (Section 2.7 and Figure 7F,G) demonstrated that the “double‐pedicle pre‐vascularization” group exhibited reduced inflammatory cell infiltration compared with the traditional single‐pedicle group, which is consistent with the sequencing results. Improved vascularization is expected to mitigate inflammation through multiple mechanisms. By providing a more robust and stable blood supply, the double‐pedicle strategy enhances oxygen and nutrient delivery, thereby alleviating tissue hypoxia—a well‐recognized driver of inflammatory signaling.^[^ ^58^, ^59^ ^]^ Consistently, animals with better vascularization showed decreased activation of inflammation‐associated pathways in transcriptomic profiling, supporting a negative correlation between vascularization and inflammation.^[^ ^60^ ^]^

Our data indeed showed that the double‐pedicle design was associated with reduced inflammatory responses compared with the single‐pedicle group. The underlying mechanisms can be explained as follows: 1) The double‐pedicle approach provides two independent vascular sources, which establish a denser and more reliable vascular network. This reduces tissue hypoxia, a well‐recognized trigger of pro‐inflammatory signaling (e.g., HIF‐1α–mediated pathways).^[^ ^61^, ^62^ ^]^ 2) By ensuring adequate perfusion, the double‐pedicle design promotes effective removal of metabolic waste products and prevents localized necrosis. Reducing necrosis limits the release of damage‐associated molecular patterns (DAMPs), thereby decreasing inflammatory cell recruitment.^[^ ^63^, ^64^ ^]^ 3) Better vascular support of the luminal surface accelerates epithelial regeneration, which restores a protective barrier. A continuous epithelial lining helps reduce direct exposure of the underlying tissue to irritants and pathogens, thus lowering inflammation. These interpretations are supported by both transcriptomic data, which showed downregulation of hypoxia‐ and inflammation‐related pathways, and histological findings, which revealed reduced inflammatory cell infiltration in the double‐pedicle group.

Despite these advances, limitations persist. Manual assembly of C‐shaped rings caused deformation from neck movements, potentially compromising mechanics; 3D bioprinting could yield integrated structures for enhanced stability. Squamous epithelium inhibited stenosis but its full metaplasia to ciliated columnar cells requires longer‐term studies or in vitro induction with growth factors. Dual‐pedicle vascularization, while effective cervically, may scale poorly for thoracic defects or larger volumes; combining with muscle flaps or angiogenic factors could accelerate ingrowth. Although CK8 upregulation suggests epithelial transition toward an airway phenotype, our study lacks definitive validation of functional maturation. Future work incorporating markers such as FOXJ1 (for ciliated cells) and MUC5AC (for goblet cells), as well as ultrastructural analysis, will be necessary to confirm ciliary differentiation and mucociliary function. Another limitation of this study is the absence of long‐term follow‐up, leaving unresolved whether the regenerated epithelium undergoes squamous metaplasia and whether the vascular network remains stable; future extended studies are needed to address these critical translational questions. Future single‐cell RNA sequencing could elucidate cell‐specific cross‐talk, optimizing cellular sources (e.g., induced pluripotent stem cells) for personalized grafts.^[^ ^65^ ^]^ Preclinical large‐animal models and clinical trials could validate scalability, paving the way for transformative solutions in airway reconstruction. From a translational perspective, several practical considerations must be addressed before clinical application. The dual‐pedicle approach, while effective in our model, increases surgical complexity and requires advanced microsurgical skills. In addition, pedicle length may be a limiting factor in humans, necessitating alternative strategies such as vascular graft interposition or selection of longer pedicle donor sites. Finally, donor site choice is critical, and options such as pectoralis major, intercostal, or omental flaps may provide reliable vascular sources with acceptable morbidity, thereby broadening the applicability of this technique in clinical practice.

Overall, this study establishes a molecular benchmark for tracheal regeneration, demonstrating that dual‐pedicle pre‐vascularization preempts pathological cascades, enabling functional integration. The most distinctive strength of this study lies in its integrated design, which simultaneously addresses the three fundamental pillars required for long‐segment tracheal reconstruction—structural support by C‐shaped cartilage, functional coverage by squamous epithelium, and sustained perfusion through a dual‐pedicle blood supply. Importantly, these components are not independent but act synergistically, with robust vascularization supporting epithelial survival and epithelial coverage protecting the underlying cartilage. Moreover, the methodology is technically straightforward and reproducible, making it not only effective for tracheal repair but also potentially adaptable to other tissue engineering applications.

## 4 Conclusion

In summary, this study presents a modular pre‐vascularization strategy for constructing a biomimetic dual‐pedicle TET that effectively addresses key challenges in long‐segment tracheal reconstruction, including ischemia, structural instability, and epithelial deficiency. By independently pre‐vascularizing autologous cartilage and skin tubes in vivo, we established robust external blood supplies that, upon integration, formed a comprehensive dual‐pedicle vascular network capable of alleviating hypoxia and inflammation. The incorporation of an autologous squamous epithelial lining further promoted luminal patency by restoring barrier function and preventing stenosis. The combined use of a stable C‐shaped cartilage framework, functional epithelium, and dual‐pedicle perfusion facilitated the coordinated maturation of cartilage, epithelialization, and vascularization. This synergistic approach resulted in enhanced biomechanical integrity, sustained airway patency, and significantly improved survival in a rabbit orthotopic tracheal transplantation model compared to single‐pedicle controls. Transcriptomic and immunofluorescence analyses confirmed these outcomes by demonstrating a molecular shift from pathological responses toward regenerative pathways. Collectively, this multifaceted strategy offers a promising and clinically translatable platform for advanced long‐segment tracheal reconstruction.

## 5 Experimental Section

### Isolation and Culture of Rabbit Auricular Chondrocytes

Healthy male New Zealand White rabbits (aged 2–2.5 months) were obtained from Jiagan Biotechnology Co., Ltd. (Shanghai, China). All animal experiments were approved by the Ethics Committee of Shanghai Pulmonary Hospital (K25‐486Y, Shanghai, China). Rabbits were anesthetized via ear vein injection of 3% sodium pentobarbital (30 mg kg^−1^; Sigma–Aldrich, USA). Under aseptic conditions, auricular cartilage was harvested, and perichondrium and adherent tissues were removed. The cartilage was minced into 1 mm^3^ fragments, washed with phosphate‐buffered saline (PBS; Gibco, USA) containing 100 U mL^−1^ penicillin and 100 µg mL^−1^ streptomycin (Gibco, USA), and digested with 0.25% trypsin‐EDTA (Gibco, USA) for 30 min at 37 °C to remove non‐chondrocytic cells. After trypsin removal, fragments were digested in DMEM/F12 medium (Gibco, USA) with 0.2% type II collagenase (Worthington Biochemical, USA) for 6 h at 37 °C in 5% CO_2_. The cell suspension was filtered through 70 µm strainers (Falcon, USA), centrifuged, washed, and resuspended in complete medium (DMEM/F12 with 10% fetal bovine serum [FBS; Gibco, USA], 1% penicillin‐streptomycin, and 50 µg mL^−1^ L‐ascorbic acid [Sigma–Aldrich, USA]). Chondrocytes were seeded at 1 × 10^5^ cells cm^−2^ and cultured at 37 °C in 5% CO_2_, with medium changes every 2–3 days. Cells were passaged at 80–90% confluence using 0.25% trypsin‐EDTA, and passages 2–3 (P2–P3) were used to avoid dedifferentiation.

### In Vitro Cultivation of C‐shaped Cartilage

12% DWJMA hydrogels were synthesized as previously described.^[^ ^20^ ^]^ Chondrocytes were suspended in the hydrogel precursor at 1 × 10^8^ cells mL^−1^ and injected into a sterilized C‐shaped mold (inner diameter 6 mm, outer diameter 8 mm, height 1.8 mm, 75% circumferential). Constructs were photo‐crosslinked under 365 nm UV light for 30 s, demolded, and photographed from multiple angles using a digital SLR camera. Constructs were cultured in chondrogenic induction medium at 37 °C in 5% CO_2_, with medium changes every 3 days, for 4 or 8 weeks. After culture, constructs were washed twice with PBS, blotted dry, and photographed. Biomechanical testing was performed using a dynamic mechanical analyzer (Instron‐5542; Instron, USA) as previously described.^[^ ^20^ ^]^ Briefly, samples were compressed horizontally or vertically at 1 mm min^−1^ until failure; elastic modulus was calculated from the 0–20% strain–stress curve. Tensile testing was conducted at 0.5 mm min^−1^ until failure, with fracture force defined as peak load. Native rabbit tracheal cartilage rings served as controls. DNA, GAG, and type II collagen contents were quantified as described.^[^ ^66^ ^]^ Histological evaluation included HE, Safranin‐O, Masson's trichrome, and type II collagen immunohistochemistry staining.

### In Vivo C‐Shaped Cartilage Regeneration in Nude Mice

Chondrocyte‐loaded hydrogels (prepared as above) were implanted subcutaneously in male nude mice (aged 4–6 weeks; Shanghai SLAC Laboratory Animal Co., Ltd., China). After anesthesia and dorsal skin disinfection with iodine, a 1 cm midline incision was made, and the subcutaneous pocket was created. Constructs were implanted, and incisions were closed with 5‐0 sutures. Mice were housed under standard conditions. Constructs were retrieved at 4 or 8 weeks post‐implantation and evaluated for gross morphology, histology (HE, Safranin‐O, Masson's trichrome, type II collagen immunohistochemistry), biochemistry (DNA, GAG, total collagen), and biomechanics (compressive strength in horizontal/vertical orientations, fracture force), with native tracheal cartilage rings as controls.

### In Vivo Development of Cartilage Tube in Rabbits

In vitro engineered C‐shaped cartilage at 4 weeks were assembled around a silicone tube with 1 mm intervals to form a cartilage tube, then implanted ectopically into cervical muscle interspaces in rabbits. After 4 weeks, tubes were retrieved and assessed for gross morphology (transverse/longitudinal views), histology (HE, Safranin‐O, type II collagen and CD31 immunohistochemistry), biochemistry (GAG, type II collagen), and biomechanics (lateral/anterior–posterior compression), with native rabbit trachea as control.

### RNA sequencing of Conventional TET Grafts in Long‐Segment Tracheal Reconstruction

Conventional TETs were constructed using in vivo‐matured cartilage tubes with single vascular pedicles.^[^ ^44^ ^]^ A 1.5 cm native tracheal segment was resected and reconstructed orthotopically with the TET using 5‐0 non‐absorbable sutures. At 4 weeks post‐transplantation, central graft tissues were harvested for RNA sequencing (*n* = 3/group), with native trachea as a control. Total RNA was extracted using the RNeasy Mini Kit (Qiagen, Germany), with quality assessed via NanoDrop 2000 (Thermo Fisher, USA) and Agilent 2100 Bioanalyzer (Agilent, USA; RIN > 8.0). Libraries were prepared with the NEBNext Ultra II RNA Library Prep Kit (New England Biolabs, USA) and sequenced on Illumina NovaSeq (150 bp paired‐end). Reads were quality‐trimmed, aligned to OryCun2.0 using STAR, and quantified with featureCounts. Differential expression used DESeq2 (padj < 0.05, |log_2_(FoldChange)| > 1). GO and KEGG analyses were performed with clusterProfiler in R. GSEA used fgsea with MSigDB Hallmark sets (h.all.v7.5.1).

### Construction of Skin Tube

Auricular skin was harvested from rabbits as described.^[^ ^35^ ^]^ Briefly, skin was separated from auricular cartilage, disinfected in povidone‐iodine for 5 min, rinsed with PBS, trimmed to 18 × 19 mm, wrapped epithelially inward ≈6 mm silicone tube, and sutured with 5‐0 non‐absorbable sutures. Skin tubes were implanted into left cervical muscle interspaces for 4 weeks (+vascularization). Non‐implanted skin tubes served as controls (‐vascularization). Samples were evaluated by HE, Masson's trichrome, and CD31 immunohistochemistry. Flap thickness and microvessel density were quantified using ImageJ and analyzed in GraphPad Prism 8.

### Construction of TET with Double Vascular Pedicles

Cartilage and skin tubes, prepared ex vivo as described above, were implanted into the right and left cervical muscle interspaces of rabbits, respectively, for 4 weeks to achieve vascularization. The tubes were then re‐exposed, with unilateral vascular pedicles preserved for each. Silicone stents were removed, and the skin tube was nested into the notch of the cartilage tube to form a dual‐pedicle TET. The constructs were secured with 6‐0 non‐absorbable sutures, re‐stented with a silicone tube, and placed adjacent to the native trachea for 2 weeks to facilitate integration. At 2 weeks post‐implantation, constructs were retrieved and evaluated for gross morphology (transverse and longitudinal views), histology (HE, Safranin‐O, type II collagen, and CD31 immunohistochemistry), biochemical composition (GAG and collagen II contents), and biomechanical properties (compressive strength in lateral and anterior–posterior orientations), with native rabbit trachea serving as the control. To validate epithelial tissue integrity, samples from the dual‐pedicle TET and cartilage tube were subjected to immunofluorescence staining for pan‐cytokeratin (a broad‐spectrum epithelial marker) and E‐cadherin (an epithelial cell adhesion molecule).

### Long‐Segment Tracheal Defect Repair with Double Vascular Pedicles

Dual‐pedicle TETs were employed for orthotopic reconstruction of long‐segment tracheal defects in rabbits (*n* = 10 per group). Briefly, the silicone stent was removed from the dual‐pedicle TET, a 1.5 cm segment of native trachea was excised, and the TET was anastomosed orthotopically using 5‐0 non‐absorbable sutures. Single‐pedicle TETs served as controls, prepared by nesting the ex vivo skin tube into the notch of the ex vivo cartilage tube (both prepared as described above), followed by implantation into the left cervical muscle interspace for 4 weeks. Upon re‐exposure, one vascular pedicle was preserved, and the construct was used for orthotopic reconstruction. Postoperative care included administration of penicillin and meloxicam for 5 days. Animals in both groups were monitored daily for signs of respiratory distress, with euthanasia performed if necessary. Lateral neck X‐rays (70 kVp, 300 mA, 0.03 s exposure) were acquired at 2 and 4 weeks post‐transplantation and analyzed using ImageJ software to calculate tracheal patency: (minimum reconstructed diameter [β] / average native diameter [α]) × 100%. At euthanasia or 4 weeks post‐transplantation, grafts (including 0.5 cm of adjacent native trachea at each end) were harvested, photographed, and evaluated for mucous obstruction. Samples were subjected to histological analysis (HE, Safranin‐O, type II collagen, and CD31 immunohistochemistry) and biomechanical testing (compressive strength in horizontal and vertical orientations; fracture force), with native trachea as control.

At 4 weeks post‐transplantation, single‐ and dual‐pedicle TET grafts were further analyzed by immunofluorescence. Bacterial infiltration was assessed using FISH staining, with sections examined under light microscopy. Apoptosis was evaluated via TUNEL assay (In Situ Cell Death Detection Kit, Roche, Switzerland) per the manufacturer's instructions. Inflammation was quantified by immunofluorescence staining for TLR4 (Abcam, UK) and MPO (Abcam, UK), markers of inflammatory signaling and neutrophil infiltration, respectively. Epithelial barrier integrity was examined by immunofluorescence staining for Claudin‐1 (Abcam, UK), a tight junction protein, and β‐defensin 1 (Abcam, UK), an antimicrobial peptide. Sections were incubated with primary antibodies overnight at 4 °C, followed by fluorochrome‐conjugated secondary antibodies for 1 h at room‐temperature. Nuclei were counterstained with DAPI (Sigma–Aldrich, USA). Images were acquired using a fluorescence microscope (Leica, Germany). Fluorescence intensities for TLR4, MPO, Claudin‐1, and β‐defensin 1 were quantified using ImageJ software (NIH, USA).

To evaluate airway epithelial differentiation of skin‐derived epithelium in transplanted grafts, immunofluorescence staining for CK14 (skin epithelium‐specific marker) and CK8 (airway epithelium‐specific marker) was performed on single‐ and dual‐pedicle TETs at 0, 2, and 4 weeks post‐transplantation. CK8 fluorescence intensity was quantified using ImageJ software for both groups at these time points.

### RNA Sequencing of Transplanted Grafts

At 4 weeks post‐transplantation, central tissues from single‐ and dual‐pedicle TETs and native trachea were harvested (*n* = 3/group). RNA extraction, library preparation, sequencing, and bioinformatics were performed as described above for conventional TETs.

### Statistical Analysis

All statistical analyses were performed using GraphPad Prism version 8.0. Data are presented as mean ± standard deviation (SD) unless otherwise specified. Sample sizes were determined based on feasibility in this rabbit model and consistency with previous studies of tissue‐engineered trachea, rather than a priori power analysis. This was acknowledged as a limitation. For comparisons among multiple groups, one‐way analysis of variance (ANOVA) was conducted, followed by Tukey's honestly significant difference (HSD) test for post‐hoc multiple comparisons. A *p*‐value < 0.05 was considered statistically significant.

## Conflict of Interest

The authors declare no conflict of interest.

## Supporting information



Supporting Information

## Data Availability

The data that support the findings of this study are available from the corresponding author upon reasonable request.
