# Supplementary material for: Dual‐Pedicle Tissue‐Engineered Trachea Promotes Biomimetic Cartilaginous Framework, Vascularization, and Epithelial Lining for Long‐Segment Tracheal Reconstruction
Source: Adv Sci (Weinh). 2025 Oct 13;12(47):e14724. doi: 10.1002/advs.202514724 (PMC12713032; doi:10.1002/advs.202514724)
Supplement: Supplementary file 1 — Supporting Information [file ADVS-12-e14724-s001.docx]

**Supporting Information**

**Dual-Pedicle Tissue-Engineered Trachea Promotes Biomimetic Cartilaginous Framework, Vascularization, and Epithelial Lining for Long-Segment Tracheal Reconstruction**

*Ziming Wang^1a^, Yuming Wang^2a^, Zihao Chen^1,3a^, Erji Gao^1a^, Liang Guo^1^, Juanjuan Li^4^, Siqiang Zheng^1^, Jiaoyu Yi^5^, Zhe-Sheng Chen^6^, Bo Tao^1*^*

^1^ Department of Thoracic Surgery, Shanghai Pulmonary Hospital, Tongji University, School of Medicine, Shanghai, China

^2^ Shanghai Lung Cancer Center, Shanghai Chest Hospital, Shanghai Jiao Tong University, School of Medicine, Shanghai, China

^3^ Department of Minimally Invasive Thoracic Surgery Center, Second Affiliated Hospital of Naval Medical University, Shanghai, China

^4^ Department of Medical Oncology, Shanghai Pulmonary Hospital and Thoracic Cancer Institute, Tongji University, School of Medicine, Shanghai, China

^5^ Department of Plastic Surgery, Renji Hospital, Shanghai Jiaotong University, School of Medicine, Shanghai, China

^6^ Department of Pharmaceutical Science, College of Pharmacy and Health Science, St John’s University, Queens, New York, United States of America

^a^ These authors contributed equally to this work.

*Corresponding to: Dr. Bo Tao, Email: tbo0820@163.com.


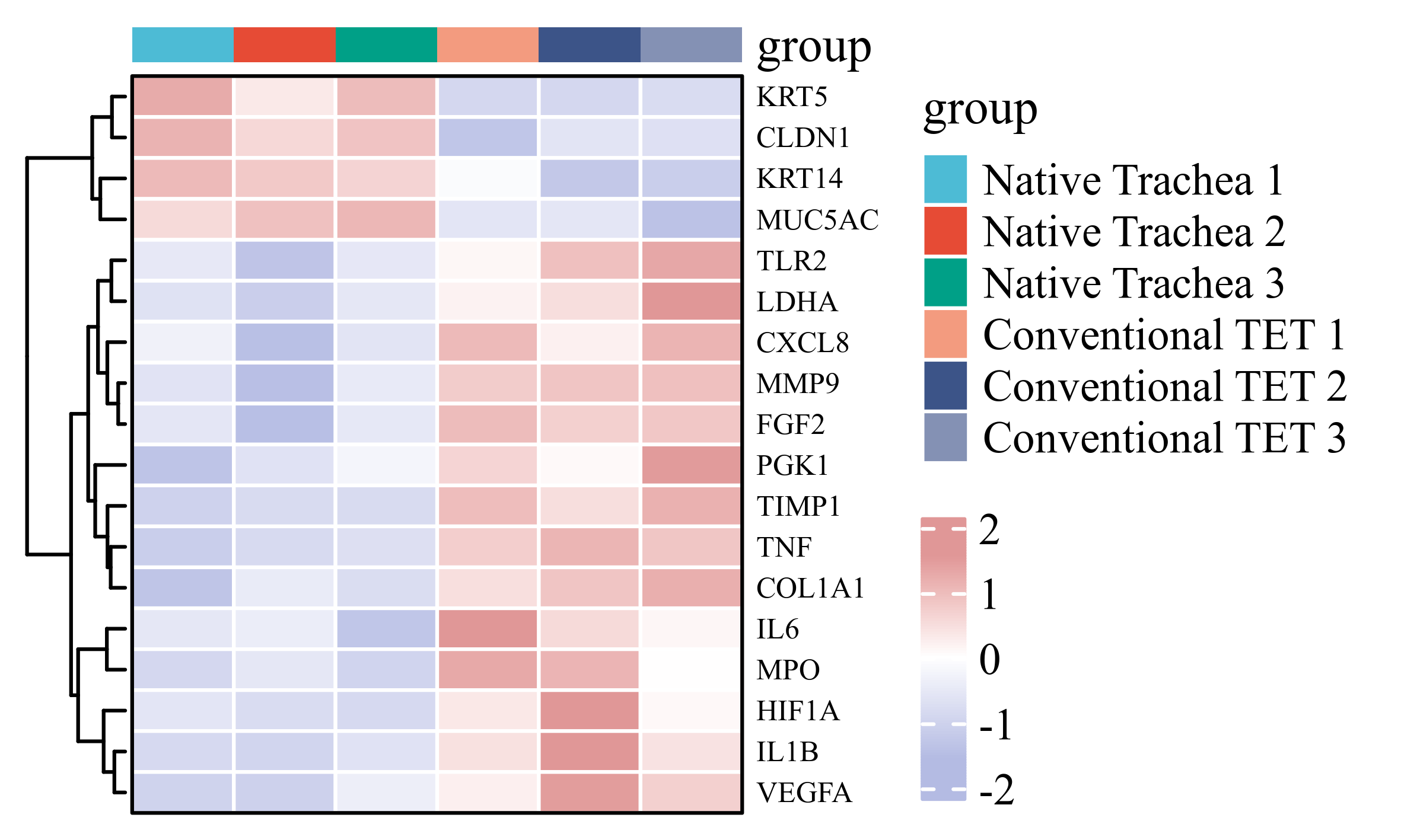


**Figure S1. Comparative Heatmap Analysis Elucidates Pathological State of Conventional TETs.** Heatmaps illustrating the expression profiles of genes associated with key biological processes, comparing conventional TET grafts at 4 weeks post-orthotopic tracheal reconstruction with native tracheas (n=3).


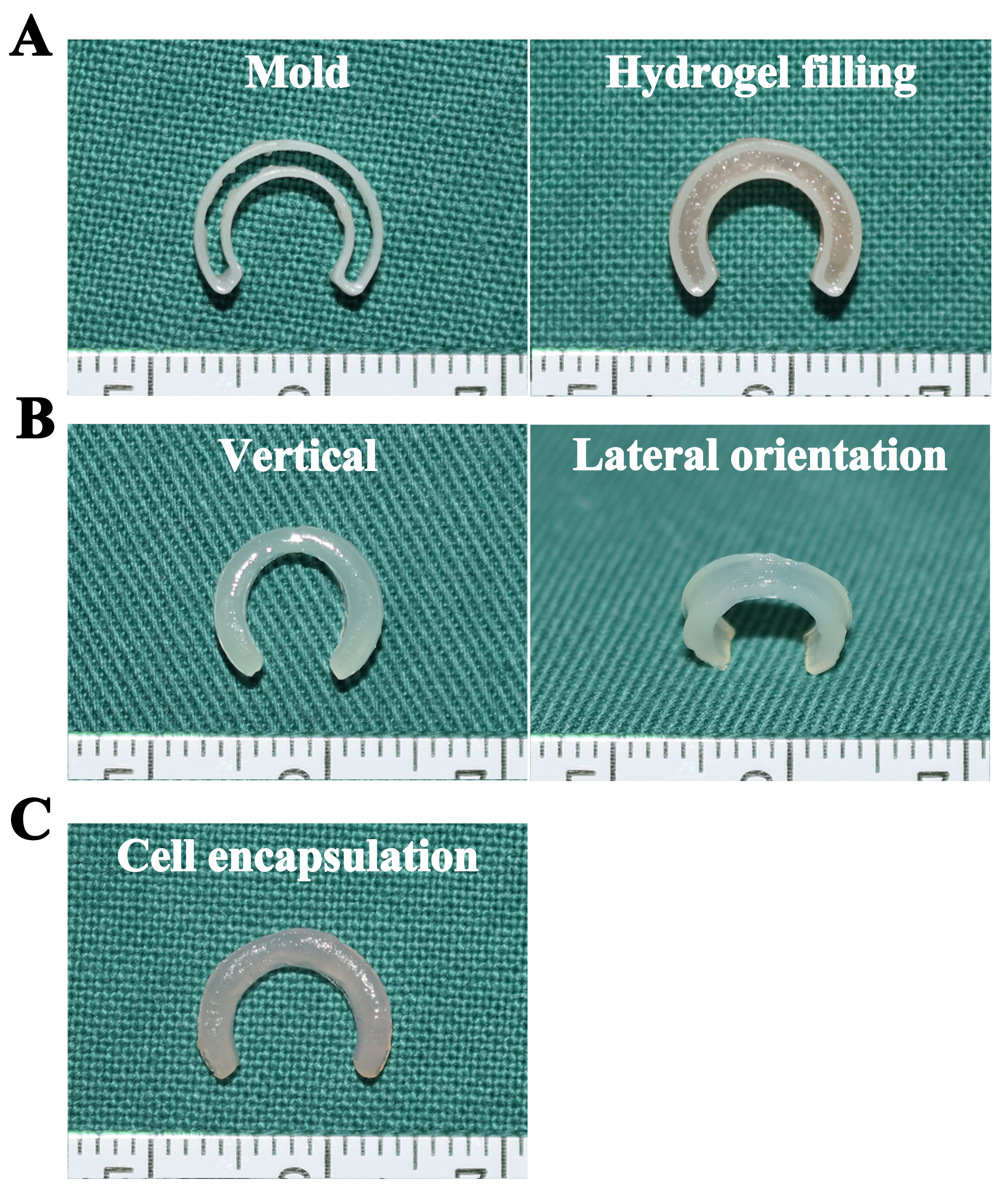


**Figure S2. Fabrication of Cell-Laden, C-Shaped Hydrogel Constructs for Cartilage Engineering.** (A) A custom-designed, C-shaped mold filled with photo-crosslinkable DWJMA hydrogel precursor solution. (B) Post-photo-crosslinking, a stable, acellular C-shaped hydrogel construct is formed, precisely replicating the mold’s geometry, as depicted in vertical and lateral views. (C) For cartilage engineering, autologous chondrocytes are uniformly suspended in the hydrogel precursor solution prior to casting, yielding a cell-laden construct with homogeneously distributed cells, primed for subsequent culture and maturation into a C-shaped cartilage module.


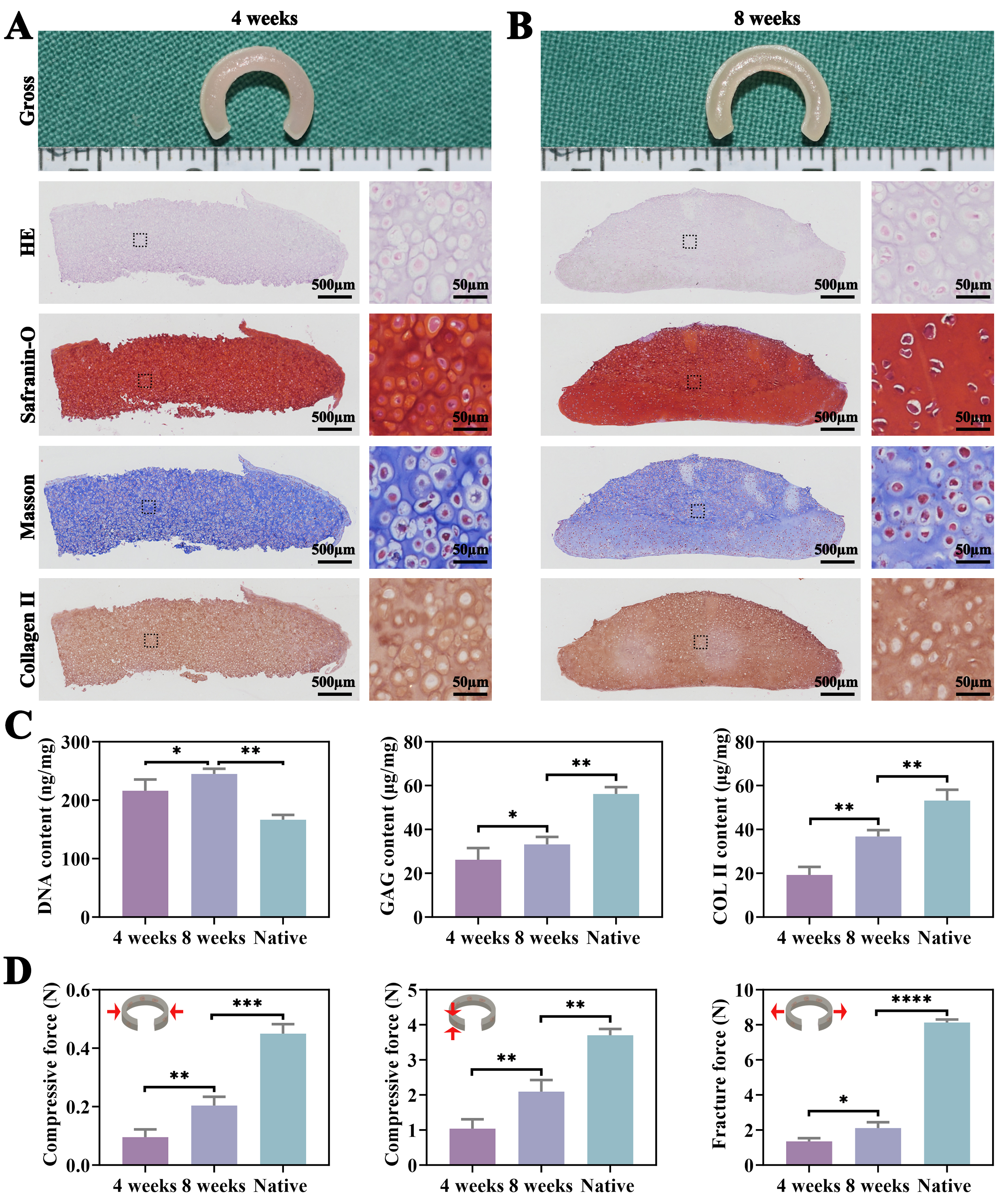


**Figure S3. Progressive *In Vitro* Maturation of Engineered C-Shaped Cartilage, Yet Inferior to Native Trachea.** Histological analysis at 4 weeks (A) and 8 weeks (B) reveals progressive matrix maturation of C-shaped cartilage constructs (HE staining), with increasing intensity of GAGs (Safranin-O and Masson's Trichrome staining) and type II collagen (immunohistochemistry), key components of hyaline cartilage. (C) Quantitative biochemical analysis confirms progressive increases in DNA content, GAG collagen, and type II collagen from 4 to 8 weeks, though 8-week constructs remain inferior to native tracheal cartilage rings. (D) Biomechanical testing shows significant improvements in compressive strength (horizontal and vertical orientations) and fracture strength over time, yet 8-week constructs exhibit inferior mechanical properties compared to native tracheal cartilage rings. Data are presented as mean ± SD (n = 5 per group). **P* < 0.05, ***P* < 0.01, ****P* < 0.001, *****P* < 0.0001.


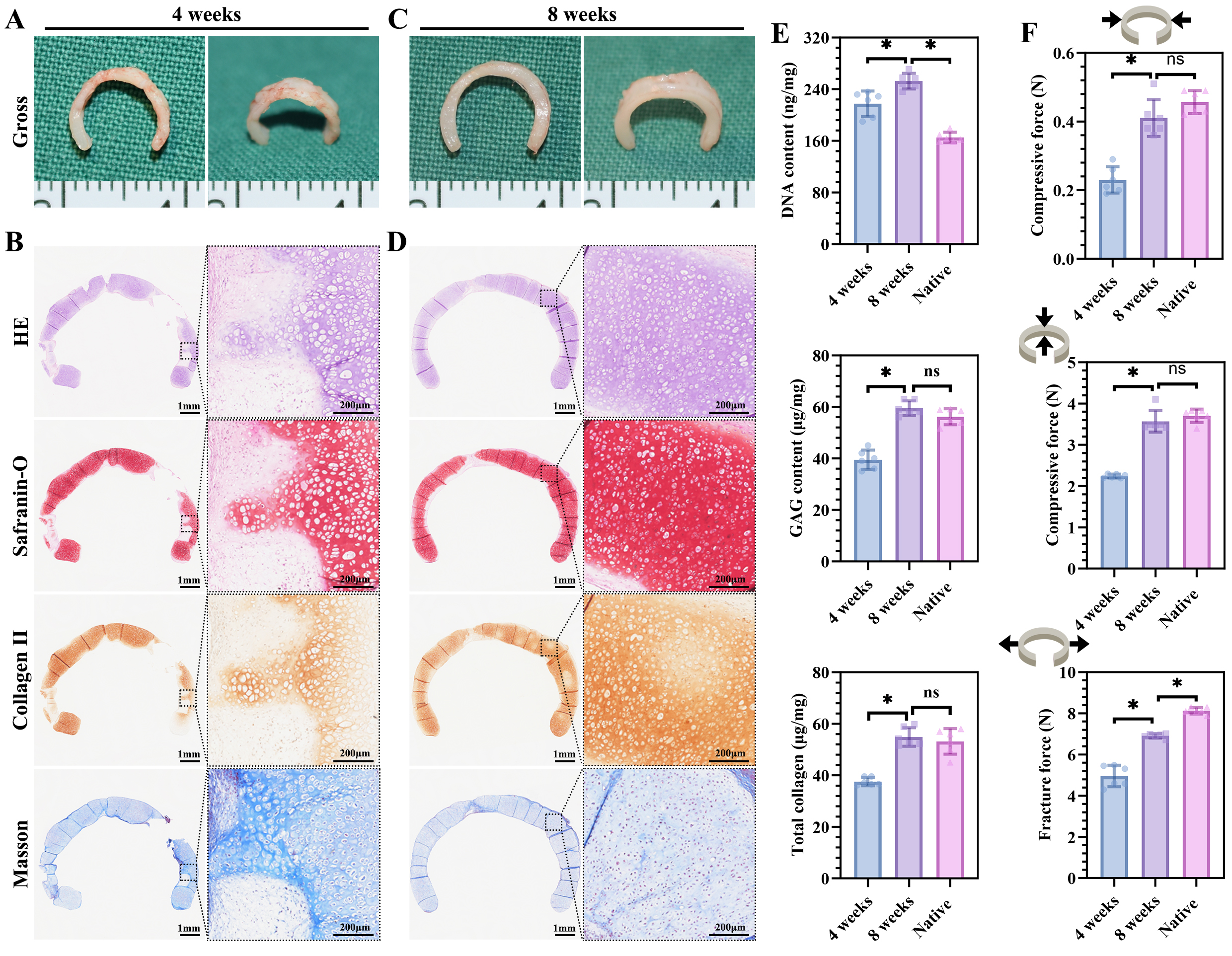


**Figure S4. Chondrocyte-Laden Hydrogel Achieve Stable C-Shaped Cartilage Maturation in Nude Mice.** To evaluate the chondrogenic potential of cell-laden hydrogels *in vivo*, chondrocyte-loaded hydrogels were implanted subcutaneously in nude mice and assessed at 4 and 8 weeks. (A, C) Gross morphology and (B, D) histological analysis (HE staining) demonstrate robust C-shaped cartilage formation and progressive maturation, evidenced by increasingly dense and organized ECM deposition, with intense staining for GAGs (Safranin-O and Masson's Trichrome staining) and type II collagen (immunohistochemistry) at 8 weeks. (E) Quantitative biochemical analysis confirms significant increases in GAG and total collagen contents from 4 to 8 weeks, approaching levels of native tracheal cartilage rings. (F) Biomechanical testing reveals significant improvements in compressive strength (horizontal and vertical orientations) and fracture resistance over time, with 8-week constructs achieving properties comparable to native tracheal cartilage rings. Data are presented as mean ± SD (n = 5 per group). **P* < 0.05, ns indicates no statistical significance.


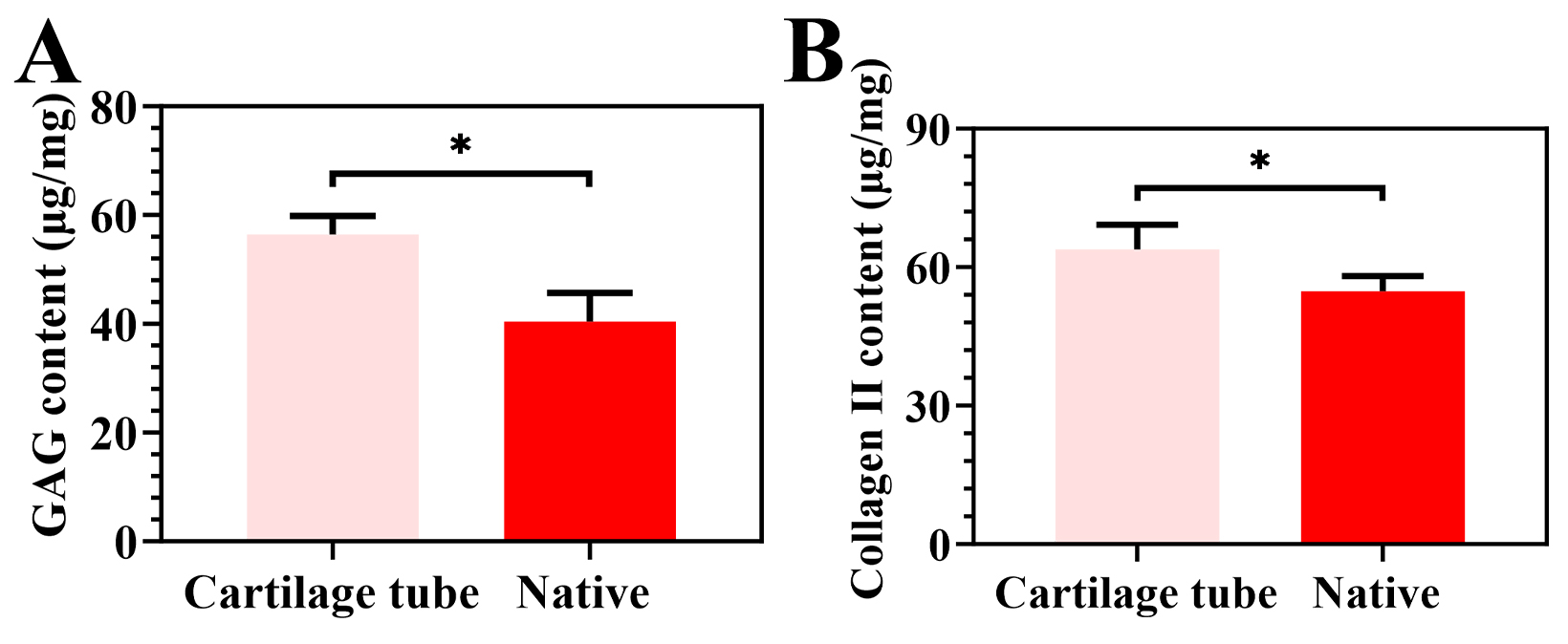


**Figure S5. *In Vivo* Engineered Cartilage Tube Exhibits a Biomimetic Biochemical Profile.** Biochemical composition of the cartilage tube was quantified after 4 weeks of *in vivo* maturation to validate matrix development. Analysis confirms robust deposition of mature ECM, with elevated levels of GAGs (A) and type II collagen (B) compared to native trachea, providing quantitative evidence that pre-vascularization promotes a biochemically mature cartilaginous framework. Data are presented as mean ± SD (n = 5 per group, **P* < 0.05).


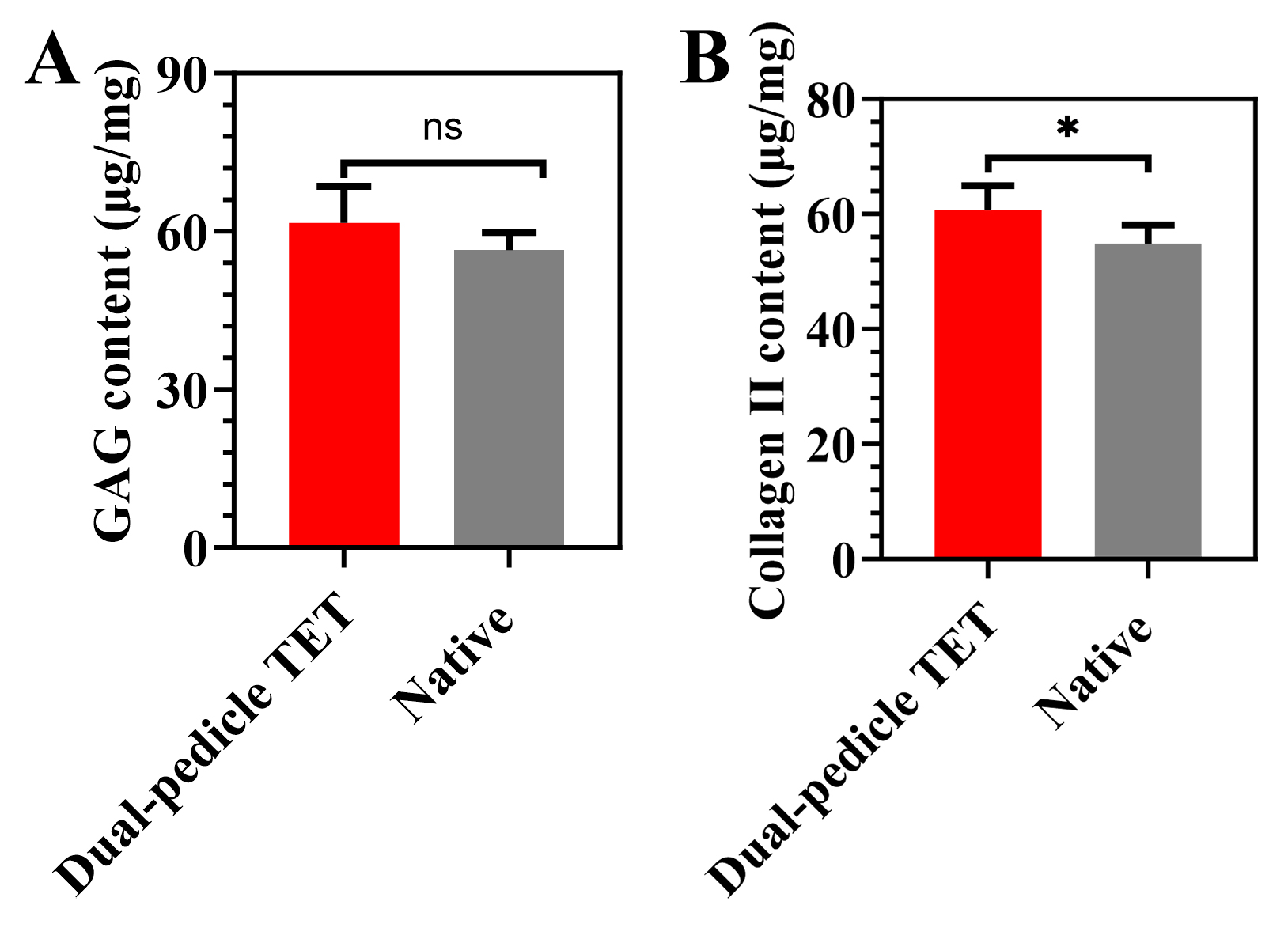


**Figure S6. Assembled Dual-Pedicle TET Maintains a Mature Cartilage Matrix.** Biochemical analysis of the fully assembled dual-pedicle TET was conducted to confirm the integrity of its cartilaginous matrix. Quantification reveals robust deposition of GAGs (A) and type II collagen (B), with levels significantly higher than those in native tracheal cartilage. Data are presented as mean ± SD (n = 5 per group, **P* < 0.05, ***P* < 0.01, ns indicates no statistical significance).


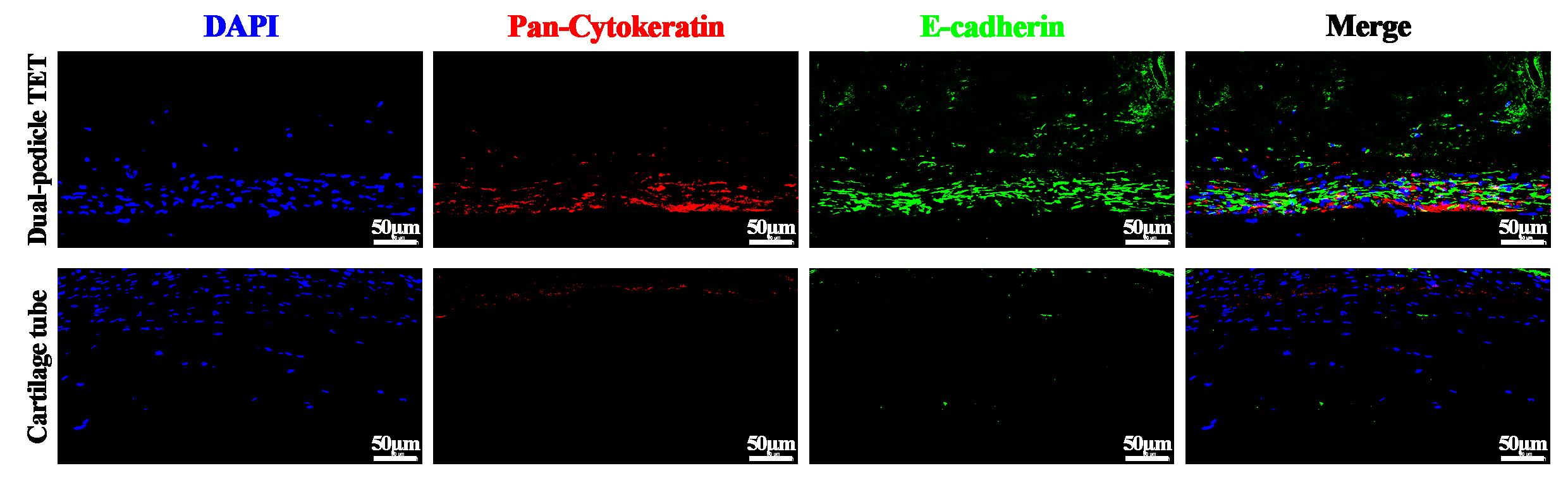


**Figure S7.** **Immunofluorescence Staining Confirms** **Epithelial Tissue Maintenance in Dual-Pedicle TET.** Immunofluorescence staining for the broad-spectrum epithelial marker of Pan-Cytokeratin and the epithelial cell adhesion molecule of E-cadherin in the dual-pedicle TET and cartilage tube groups.


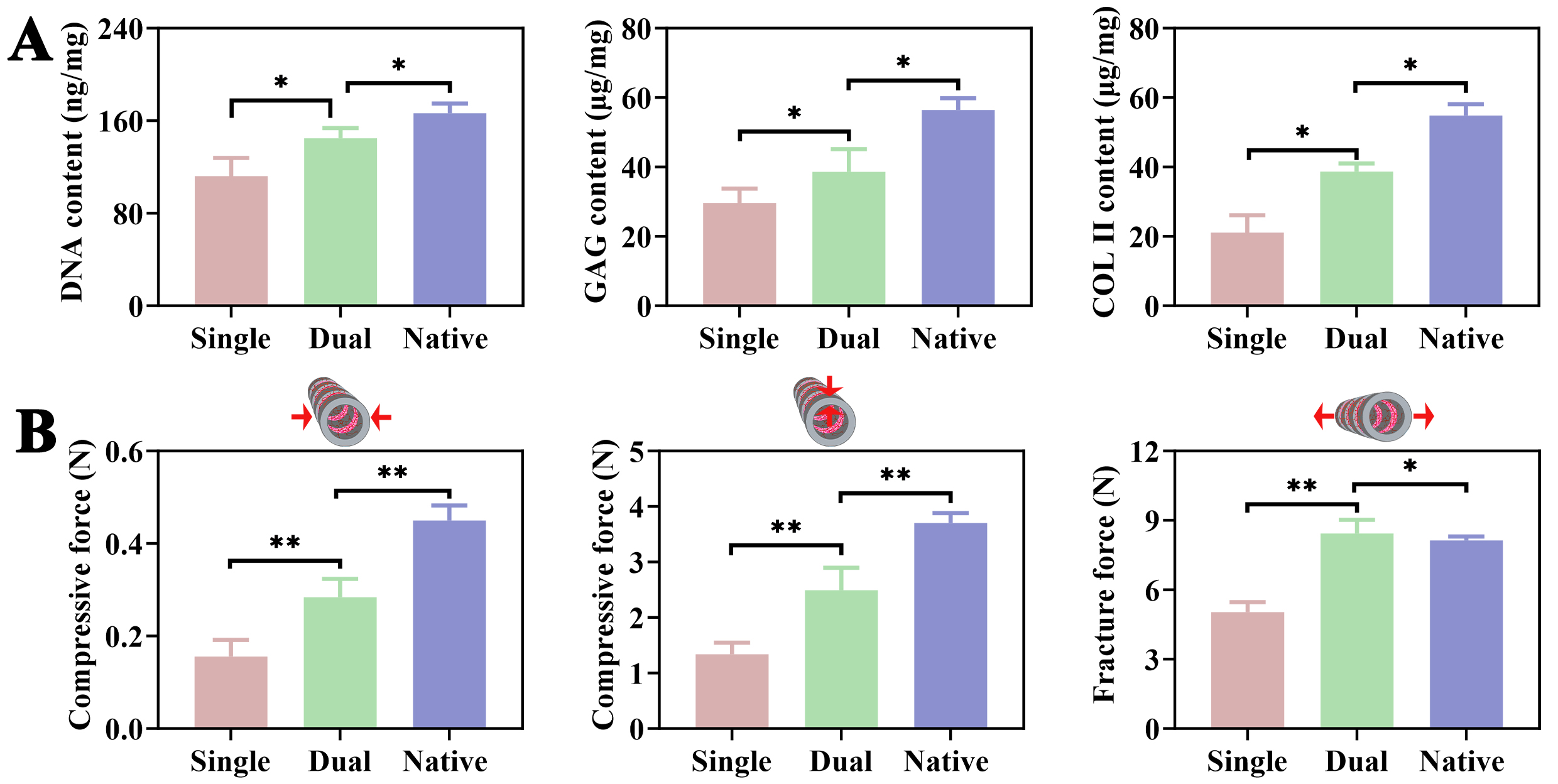


**Figure S8. Dual-Pedicle Strategy Maintains Biochemical and Mechanical Integrity of Grafts Post-Transplantation.** Biochemical and biomechanical properties of grafts were evaluated at 4 weeks post-transplantation to support histological findings. (A) Biochemical analysis shows that dual-pedicle TETs retain significantly higher levels of DNA, GAGs, and type II collagen compared to single-pedicle grafts. (B) Biomechanical assessment demonstrates superior compressive strength (lateral and anterior–posterior orientations) and fracture resistance in dual-pedicle grafts compared to single-pedicle grafts. Data are presented as mean ± SD (n = 5 per group). **P* < 0.05, ***P* < 0.01.


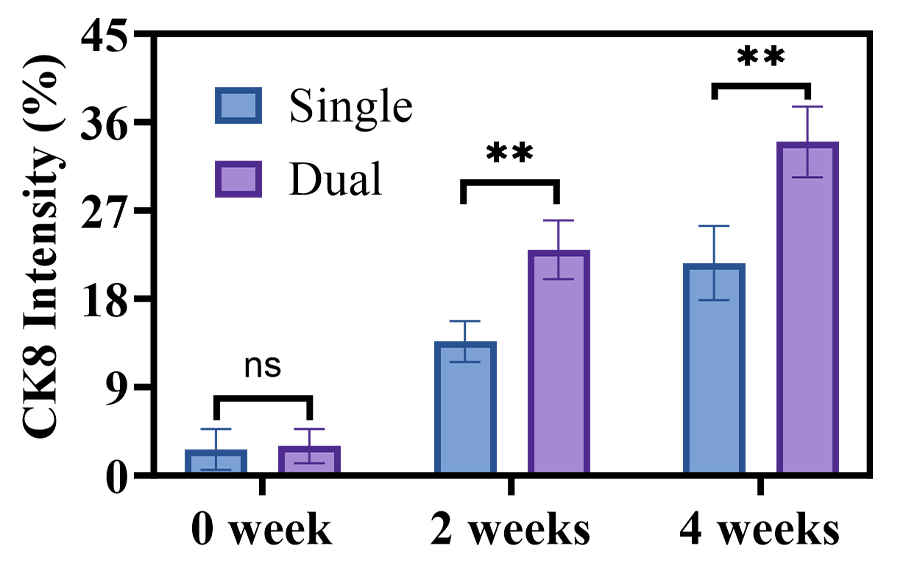


**Figure S9. Quantitative analysis of CK8 fluorescence intensity in the single-pedicle TET and dual-pedicle TET groups at 0, 2, and 4 weeks.** Data are presented as mean ± SD (n = 5 per group). **P < 0.01, ns indicates no statistical significance.


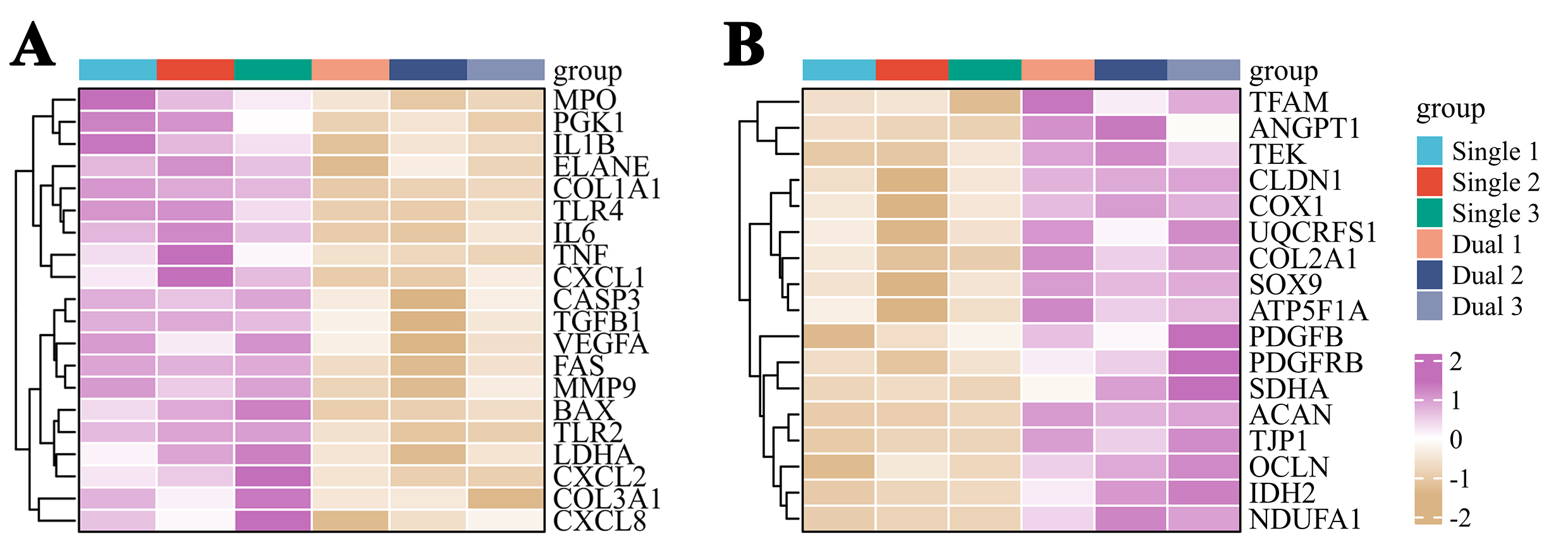


**Figure S10. Opposing Gene Expression Signatures Distinguish Single-Pedicle and Dual-Pedicle Graft Outcomes.** Detailed heatmaps visualizing expression profiles of key gene sets from transcriptomic analysis. (A) The single-pedicle group exhibits elevated expression of genes associated with hypoxia, inflammation, and fibrotic remodeling. (B) In contrast, the dual-pedicle group, closely resembling native trachea, shows high expression of genes critical for maintaining tissue structure (e.g., epithelial barrier) and healthy cellular function (e.g., aerobic metabolism).
